# Atlas-Guided Cluster Analysis of Large Tractography Datasets

**DOI:** 10.1371/journal.pone.0083847

**Published:** 2013-12-30

**Authors:** Christian Ros, Daniel Güllmar, Martin Stenzel, Hans-Joachim Mentzel, Jürgen Rainer Reichenbach

**Affiliations:** 1 Medical Physics Group, Institute of Diagnostic and Interventional Radiology I, Jena University Hospital - Friedrich Schiller University Jena, Jena, Germany; 2 Pediatric Radiology, Institute of Diagnostic and Interventional Radiology I, Jena University Hospital - Friedrich Schiller University Jena, Jena, Germany; Universiteit Gent, Belgium

## Abstract

Diffusion Tensor Imaging (DTI) and fiber tractography are important tools to map the cerebral white matter microstructure in vivo and to model the underlying axonal pathways in the brain with three-dimensional fiber tracts. As the fast and consistent extraction of anatomically correct fiber bundles for multiple datasets is still challenging, we present a novel atlas-guided clustering framework for exploratory data analysis of large tractography datasets. The framework uses an hierarchical cluster analysis approach that exploits the inherent redundancy in large datasets to time-efficiently group fiber tracts. Structural information of a white matter atlas can be incorporated into the clustering to achieve an anatomically correct and reproducible grouping of fiber tracts. This approach facilitates not only the identification of the bundles corresponding to the classes of the atlas; it also enables the extraction of bundles that are not present in the atlas. The new technique was applied to cluster datasets of 46 healthy subjects. Prospects of automatic and anatomically correct as well as reproducible clustering are explored. Reconstructed clusters were well separated and showed good correspondence to anatomical bundles. Using the atlas-guided cluster approach, we observed consistent results across subjects with high reproducibility. In order to investigate the outlier elimination performance of the clustering algorithm, scenarios with varying amounts of noise were simulated and clustered with three different outlier elimination strategies. By exploiting the multithreading capabilities of modern multiprocessor systems in combination with novel algorithms, our toolkit clusters large datasets in a couple of minutes. Experiments were conducted to investigate the achievable speedup and to demonstrate the high performance of the clustering framework in a multiprocessing environment.

## Introduction

Diffusion Weighted Imaging (DWI) [1] has been around for more than two decades in the MR imaging community and has become a well-established Magnetic Resonance Imaging (MRI) technique that measures the translational displacement of water molecules in biological tissue, also known as Brownian motion.

Diffusion Tensor Imaging (DTI) exploits this effect and facilitates the estimation of diffusion tensors that enable the extraction of quantitative measures such as diffusivity, apparent diffusion coefficient or Fractional Anisotropy (FA). The resulting voxel-wise diffusivity profiles are thus potential indicators for the underlying microstructural axonal pathways in the brain [2]. In order to approximate these white matter structures [3], [4], fiber trajectories can be reconstructed using various tractography techniques [5]–[10].

For whole brain tractography, the reconstructed datasets contain a wealth of information and consist of several thousand up to more than one million streamlines. Though such datasets approximate the underlying brain structure in high detail, the fiber tracts (i.e. streamlines) have no apparent structural organization and are loosely distributed throughout the brain. Hence, it is unclear to which underlying white matter structure particular fiber tracts belong and if tracts are part of either the same or of distinct structures.

Even though fiber tracts are often color-coded according to their spatial orientation, this coloring is mainly a visual aid that does not help to decipher the structural organization of the tractography datasets. However, various potentially useful applications would greatly benefit from disentangling the loosely organized fiber tracts. Fiber tracts, grouped into meaningful bundles that represent the underlying white matter structures correctly, are useful for the assessment of structural connectivity between distinct brain regions [11] or for determining structural integrity of distinct white matter pathways.

Correct assignment of fiber bundles may also be helpful for the assessment of tumors and delineation of tumorous tissue, as this may aid to determine if white matter bundles have been infiltrated by the tumor or whether the bundles have merely been dislocated [12], [13]. The incorporation of such fiber bundle specific information (e.g. course, spatial location, integrity, etc.) into treatment planning, neuronavigation as well as radiation therapy, will aid the neurosurgeon and ultimately help the patients.

Another important area are Fiber bundle Driven Techniques (FDTs) for the quantitative analysis of structural white matter differences between groups of subjects (e.g. patients vs. healthy controls) [14]–[17]. Compared to established and predominantly applied techniques such as voxel-based morphometry [18] or tract-based spatial statistics [19], FDTs enable the analysis of individual white matter bundles. In this context, FDTs aid the quantitative analysis as they can be instrumented to prevent interpolation effects between distinct white matter structures that result from the coregistration of various datasets [17], [20].

Disentangling the structural organization of tractography datasets is of extraordinary importance for a number of potentially useful applications but lacks applicability as the division of fiber tracts into meaningful bundles is difficult. Even though the bundling of fiber tracts can be performed manually, this type of processing is prone to errors, remains highly time-consuming and an operator with fundamental neuroanatomical knowledge is essential.

Machine learning methods are auspicious techniques for the automatic extraction of fiber bundles. Classification for example is a supervised machine learning method that uses predefined prototype classes (e.g. a white matter parcellation, atlas, etc.) to predict the membership of fiber tracts to a class. With the increasing availability of atlases and parcellations [21]–[23] as well as guidelines to accomplish a reproducible segmentation of the white matter [24], atlas-based classification has become a convenient tool to define the fiber bundles that correspond to specific regions of the atlas [25].

If an atlas is not available, fully automated unsupervised learning techniques can be used instead of supervised methods. Fiber clustering is such an unsupervised method that analyzes the similarities between fiber tracts in order to assemble similar fiber tracts into distinguishable fiber bundles. While classification is only able to define fiber bundles that correspond to classes in the anatomical atlas, cluster analysis groups tracts into fiber bundles based on the similarity of distinct features of the tracts. In practice, however, the clustering of the tracts is rarely optimal. Fiber bundles are often divided into various parts or different bundles are falsely merged together. As the outcome of cluster analysis is influenced by various factors, such as the similarity measure, the clustering parameters and the data itself, it is challenging to set up the cluster analysis in a way that consistent and reproducible clustering of different datasets with a good correspondence to anatomical fiber bundles is achieved. Considering the high variability of different datasets, it is in fact unlikely that clustering without anatomical guidance can be used to extract fiber bundles reliably in such a way that the generated bundles are anatomically correct for all datasets. Hence, a consistent, reproducible and correct extraction of fiber bundles across multiple subjects solely based on tract similarity and without anatomical guidance is difficult to achieve.

One fundamental drawback of clustering is the high computational complexity, which is immanent to the majority of conventional clustering algorithms [26]. Since fiber tracts are sets of points that constitute complex trajectories in 3D space, appropriate measures are indispensable to determine the similarity between the fibers. However, both the costly clustering and the complex similarity measures increase the total computational load and typically restrict cluster analysis to small datasets that consists of only a few thousand fiber tracts. In recent years, a multitude of methods have has proposed for both classification and clustering of fiber tracts [27]–[37]. The first clustering approaches solely relied on similarity measures to group tracts into bundles (e.g. Ding et al. [27], Moberts et al. [29]). Various researchers investigated spectral clustering approaches [28], [31] and used “spectral embedding” to map the fibers to three-dimensional Euclidean space, which enabled the clustering algorithm to handle the inherent complexity of fiber tracts more easily [31]. These first fiber clustering methods primarily focused on single subject clustering and neglected anatomical information. Later on, researchers started to experiment with the clustering of multiple input datasets and the incorporation of anatomical features into the clustering. While O'Donnell et al. [38], for example, performed multi-subject clustering to create an atlas that was used to automatically label fiber tracts, Maddah developed an expectation-maximization algorithm [33] and used Bayesian modeling to integrate spatial anatomical information. More recent approaches, focused on repeated, simultaneous clustering of multiple datasets [39] and fast voxel-based clustering of rasterized tracts [40], but neglected anatomical correspondence of fibers and obtained clusters.

Overall, despite the multitude of available methods that have been proposed for both classification and clustering of fiber tracts, fast, consistent and anatomically correct clustering for multiple subjects is still challenging. To overcome these shortcomings, we present a new clustering framework that introduces the novel cluster analysis technique CATSER (Cluster Analysis Through Smartly Extracted Representatives). While conventional clustering techniques are often limited by long processing times, CATSER is characterized by low computational complexity and is applicable to large tractography datasets that contain hundreds of thousands of fiber tracts. In order to reduce the computation time, our approach relies on random sampling, partitioning of the data and parallel computing.

**Figure 1:**
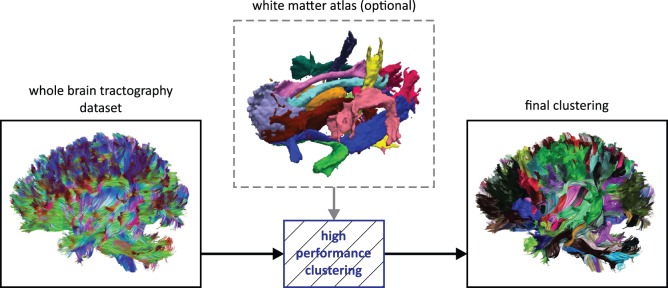
Example of whole brain fiber tractography and fiber tract clustering. Fiber tractography is performed to generate streamlines that approximate the underlying axonal pathways of the white-matter architecture (left). Tracts are color-coded according to their orientation with red = left-right, green = anterior-posterior and blue = inferior-superior. Clustering methods can be used to cluster the fiber tracts and to group similar tracts into fiber bundles or set of tracts (right). By employing a white matter atlas that consists of several white matter bundles (middle), the automatic extraction can be improved to retrieve anatomically correct fiber bundles.

Like other authors [41], we believe that hybrid techniques that combine clustering and parcellation-based (or atlas-based) classification approaches will be instrumental to move the field of automated fiber tract segmentation techniques forward. For this reason, CATSER was designed to be used in conjunction with a white matter atlas (see Figure 1) in order to achieve a more consistent extraction of fiber bundles. With such a predefined segmentation of the white matter, cluster analysis is facilitated in partitioning the tracts according to the predefined regions of the atlas. The additional anatomical information of the atlas is used to guide the clustering by influencing the formation of the clusters on the basis of spatial agreement between fiber tracts and atlas classes. If the atlas regions are defined in accordance with the underlying white matter structure, anatomically correct clustering with good correspondence to the anatomy will result.

## A Framework for the Atlas-Guided Cluster Analysis of Large Tractography Datasets

### A Note on Fiber Tracts, Similarity Measures and Notation

We assume that fiber tracts are represented by an ordered set of points in three-dimensional space with arbitrary length that consist of at least two points. Though cluster analysis in itself is not restricted to fiber tracts and can be used for grouping all kinds of objects (e.g. points, documents, etc.), we will use the term fiber tract throughout the manuscript instead of the more general term object. As the methodology of the paper is quite extensive, a glossary is provided (Glossary S1) that contains short explanations for frequently used terms and abbreviations.

Clustering techniques employ similarity measures to determine the similarity between tracts by comparing specific and distinguishable properties or features of the tracts (e.g. differences in length, orientation, etc.). We call a function that describes the similarity of two tracts 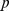 and 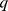 from a dataset 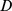 on the basis of such properties, a distance or similarity function 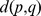 if the function is symmetric, positive semidefinite and reflexive:
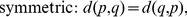
(1)

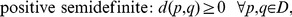
(2)

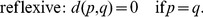
(3)


Thus, the distance between two tracts decreases the more similar the tracts are and increases in the opposed case. Throughout this theory section we use this general definition of similarity functions. For the actual clustering of a dataset, an explicit similarity function that satisfies our general definition (see above) has to be used. Explicit similarity measures that are used in this manuscript are defined in section “Similarity measures” of the appendix.

### CATSER – Cluster Analysis Through Smartly Extracted Representatives

CATSER is based on the CURE (Clustering Using REpresentatives) algorithm that was initially proposed by Guha et al. [42] for clustering huge databases. Both techniques can essentially be categorized as agglomerative hierarchical clustering methods that use an iterative bottom-up approach in which the most similar tracts are merged during each iteration.

Tractography datasets can consist of hundreds of thousands of tracts that approximate the axonal pathways in the brain. However, the number of fiber bundles that are concealed in such datasets is considerably smaller compared to the overall number of tracts. As the number of bundles per dataset is estimated to range between 140 and 500 [29], [39], it is obvious that each bundle consists of numerous tracts that capture minuscule details of the bundle. Since, the tracts in each bundle are quite similar, the dataset in itself is inherently redundant. CURE and CATSER exploit this redundancy. Instead of clustering the whole dataset as it is the case with conventional clustering methods, they process only a reduced sample and determine a set of prototype bundles. Remaining tracts of the dataset that are not part of the initial sample are then assigned to their most similar prototype clusters.

To reduce computation time and to improve the clustering results, CATSER employs random sampling, partitioning and outlier elimination. Compared to CURE, CATSER performs a modified, more outlier-sensitive clustering approach to overcome some of CURE's limitations [43]. To this end, the original algorithm was modified to incorporate Local Outlier Factors (LOFs) [44] that provide insight about the structural organization of the data. A LOF gives a rating to each tract that specifies the degree how outlying a tract is with respect to other tracts. The LOFs are used in the cluster analysis to increase the discrimination between true clusters and clusters that are presumed to be outliers.

#### Basic CATSER workflow

**Figure 2:**
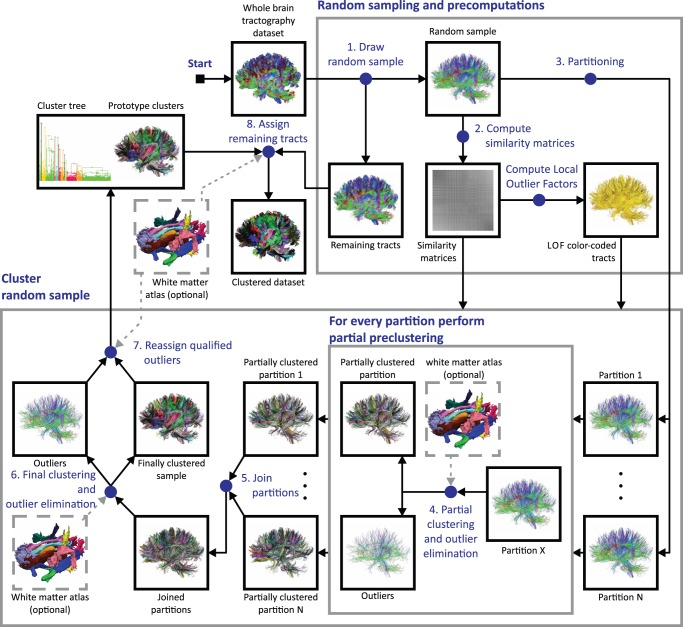
Overview of the cluster analysis technique. The size of the whole brain tractography dataset is reduced by extracting a random sample (1). For this reduced random sample, similarity matrices and Local Outlier Factors (LOFs) are computed, which are subsequently used during the clustering process (2). The reduced random sample is then automatically partitioned into a user defined number of N independent partitions (3). A first-pass partial clustering as well as outlier elimination is performed for each partition (4) before resulting clusters of all partitions are joined together (5). Resulting prototype clusters are generated during the second pass clustering (6). Outliers are then reassigned to the nearest prototype cluster in order to obtain the final clustering and the hierarchical cluster tree (7). During the last step, remaining tracts that were not part of the random sample in (1) are finally assigned to the nearest prototype cluster (8). The last two steps (step 7 and 8) are only performed for tracts that are sufficiently similar to a prototype cluster. To integrate anatomical information into the cluster analysis, a white matter atlas can be used as input during steps 4 and 6–8 (for details see section “Integration of anatomical information into the cluster analysis”).

The individual processing steps of the CATSER clustering algorithm are presented in this section and illustrated in Figure 2. In order to exploit the redundancy in the data, the whole brain tractography dataset is randomly divided into two parts (step 1): the reduced random sample and the remaining tracts that are not part of the reduced random sample. The minimum random sample size can be estimated by employing Chernoff bounds [42]. Assuming that every discernible cluster has a minimum size, the minimum reduced random sample size can then be computed so that this sample contains at least a tract fraction of each cluster with high probability. As the size of the smallest cluster in the dataset is unknown, the necessary estimation of its size limits the ability to detect smaller clusters if they exist in the dataset.

For various clustering techniques, the computation of similarities between tracts is integrated into the clustering itself. As this results in redundant computations and degraded performance of the algorithm, we separated the computation of the similarities from the cluster analysis. This greatly improves the overall performance of the clustering and circumvents not only redundant computations but enables the parallel computation of the similarities. Similarities as well as LOFs for the sample are therefore precomputed prior to the clustering (step 2).

The subsequent agglomerative hierarchical clustering procedure of CATSER is used to generate a user-defined number of clusters (details about the clustering process itself are presented in the following section “Formation of clusters”). The clustering is essentially a two-pass process, divided into a partial preclustering and a final clustering stage. In the beginning of the first stage, each tract is considered a singular cluster. The partial preclustering is then primarily a coarse grouping of the most similar clusters that reduces the number of clusters substantially. A strictly sequential processing of the data during the clustering is therefore not necessary [42]. Thus, we randomly divide the reduced random sample (step 1) into a set of partitions each of which contains approximately the same number of fiber tracts (step 3) and cluster each partition separately (step 4). In order to speed up the cluster formation during the first pass, the clustering of the separate partitions can be performed in parallel. After this first pass, the preclustered data of the partitions are joined together (step 5). With this set of joined clusters, the final clustering stage is performed and prototype clusters are generated (step 6).

During both clustering stages (steps 4 and 6), outlying tracts are identified and removed from the dataset. Due to the shuffling and separation of the data into multiple partitions, tracts of a cluster will be scattered across partitions and tracts may be unintentionally labeled as outliers during the clustering, even if a multitude of similar tracts are situated nearby but are placed in other partitions. To warrant that outlying tracts are true outliers, tracts that were previously labeled as outliers are reevaluated and assigned to the nearest prototype cluster (step 7) if the similarity between tracts and cluster is sufficiently high (for details see section “Assigning and reassigning tracts”). In the final step, remaining tracts that are not part of the reduced random sample (step 1) and have not been appointed to a cluster yet, are also assigned to the nearest prototype cluster (step 8) if the similarity between cluster and tracts is high (for details see section “Assigning and reassigning tracts”).

During the whole clustering process, a hierarchical cluster tree that contains all successive merging steps is generated. The cluster tree enables not only the visualization of the individual clustering steps with dendrograms, but also the retrospective extraction of bundles or a subset of tracts from the bundles.

#### Formation of clusters

Conceptually, CATSER employs agglomerative hierarchical clustering during both clustering stages (steps 4 and 6 in Figure 2). Starting with a set of clusters, the iterative clustering process is performed until certain stopping criteria are satisfied (e.g. a user-defined number of clusters is reached). In each iteration of the clustering, the two most similar clusters are selected and merged to form a new cluster.

In order to determine the similarity between two clusters, only a subset of tracts from each cluster is considered. This subset consists of a set of well distributed tracts that capture the geometry of the cluster and act as representative tracts. To start the selection of appropriate representatives tracts, the center of the cluster is determined by locating the cluster medoid. For a cluster 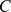 with 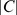 tracts, the medoid 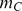 is defined as the tract in 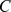 whose average distance or dissimilarity to all tracts in 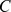 is minimal:
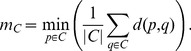
(4)


**Figure 3:**
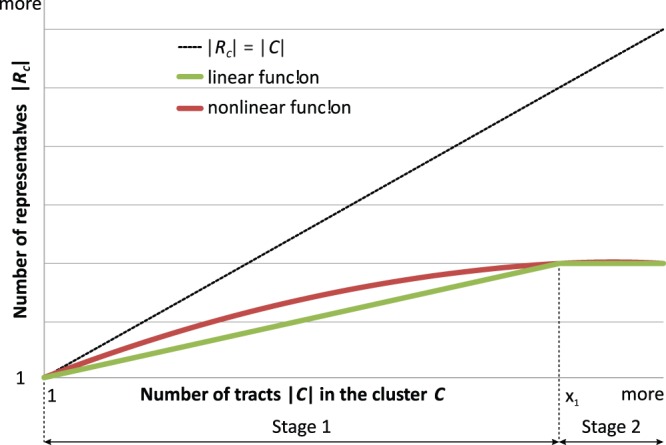
Determination of the number of cluster representatives. To determine the number of representatives 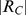 for a cluster 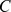 we use a two stage approach. As long as the number of tracts 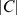 in cluster 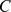 is smaller than 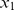, 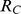 is selected in dependency of 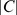 using either a linear or a nonlinear function (stage 1). If 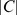 is larger than 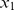, 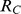 is set to a constant value (stage 2).

After the medoid has been identified, representative tracts are determined iteratively and added to the set of representatives 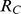. In every iteration, the tract in 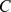 that has the maximum distance to the medoid 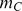 as well as to all other representatives in 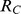 becomes a new representative and is added to 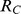. This iterative selection process guarantees that the representatives are well distributed across the cluster. An important aspect for the selection of representatives is the number of representatives that are used for the clustering. In practice, this is a trade-off between correct clustering, accurately assessing the cluster shape, achieving computational efficiency and robustness to noise. Clusters are not static, but evolve and grow. Therefore a fixed number of representatives cannot be used, as all tracts of the cluster would become a representative if the size of the cluster is smaller than the number of representatives. By reducing the ratio between number of representatives 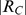 and the cluster size 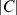 with 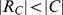, the selection algorithm can reject tracts that are outlying. In order to select 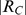, we briefly divide the cluster formation process into two stages. In the first stage, we chose 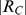 with respect to the individual size of each cluster and carefully adapt it to reflect changes in the cluster size. As the cluster size and the number of representatives increases the computational efficiency decreases due to the additional calculations that have to be performed for each additional representative. In order to maintain the computational efficiency, we define a second stage with a constant number of representatives. To select the number of representatives, we use a monotonically increasing, piecewise-defined function that resembles these two stages (see Figure 3). The function 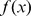 computes the number of representative 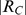 in dependency of the number of tracts in the cluster 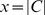:
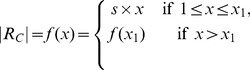
(5)with 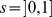 and 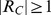 for all 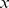.

Instead of such a linear function, nonlinear functions (e.g. monotonically increasing, interpolation functions or square root functions) might also be employed to achieve a smooth transition between the stages.

The similarity or distances between two clusters 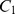 and 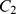 is denoted as 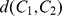 and defined to be the distance between the two most similar representatives of 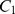 and 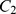. Denoting the sets of representatives for 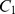 and 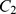 as 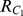 and 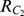, the formal definition of 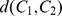 is given by:
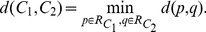
(6)


Representatives play an important role for the formation of clusters by acting as an simplification that approximates their shapes and structures. Since only representatives are used to determine the similarity between clusters, the number of comparisons and the computation time are dramatically reduced. While carefully selected representatives help to prevent negative effects of outlying tracts, such as adverse agglomerative behavior (so called chaining effects) [26], they also enable the technique to cope with clusters of arbitrary shapes. Various conventional clustering techniques (e.g., k-means) are limited to spherical cluster shapes in the similarity-based domain. By using representatives to approximate the structural organization of the cluster, the clustering method can handle arbitrarily shaped clusters correctly [42].

Throughout the clustering, outliers are handled under the assumption that they are typically isolated and do not belong to any cluster. Therefore, they do not exhibit the typical agglomerative behavior in contrast to real clusters [42]. In comparison to tracts that belong to a cluster, the neighborhoods of outliers are generally sparse and distances to other tracts of the dataset are significantly larger. Consequently, clusters that grow very slow and contain only few tracts at the end of the clustering are labeled as outliers.

#### Local Outlier Factor (LOF)

Local outlier factors [44] are a density-based approach to obtain a score for each tract that specifies its outlierness. By employing the precomputed tract-similarities, the density distribution of the tracts is analyzed to compute the LOFs. First, the k-Nearest Neighbors (k-NNs, i.e. the k-most similar tracts) are determined for each tract. The distances to these k-NNs are then utilized to compute the local tract density for each tract. With the local tract density as well as the local tract density of its k-NNs, the LOF of each tract is calculated. Practically, a LOF is an estimate on how outlying a tract is compared to its k-most similar tracts.

**Figure 4:**
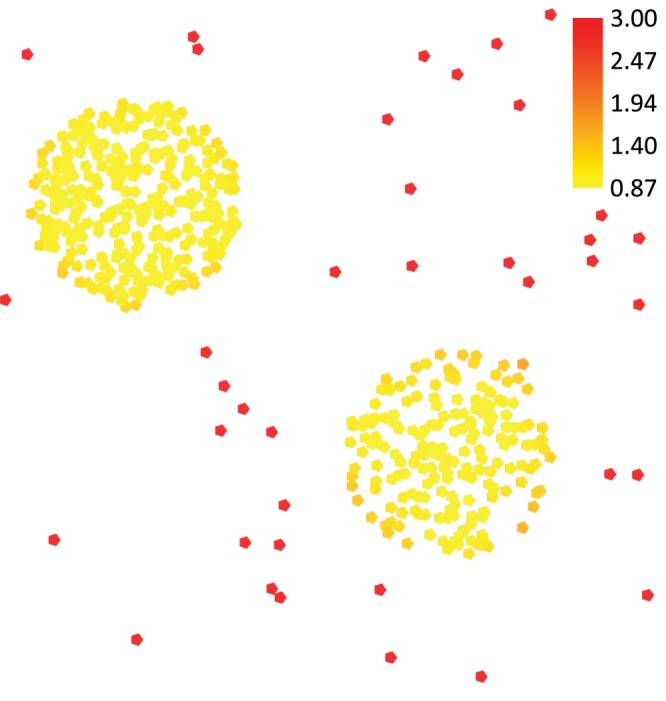
Color-coded visualization of Local Outlier Factors (LOFs) for a set of points in 2D. For the points, LOFs have been computed that indicate how outlying the points are compared to their k-most similar tracts. The points are color-coded with the values of their respective LOFs, whilst yellow denotes low and red high LOF values.

LOFs have the favorable property to specify an outlierness-rating for each tract instead of a fixed labeling that indicates whether the tract is either an outlier or not. While the LOFs capture the sparsity of the neighborhood for each tract with a single value, the upper bound of the LOFs depends on the similarities in the dataset. Tracts that lie deep inside of a cluster have a LOF that is approximately 1 or less, whereas the LOF of tracts increases the more isolated the tracts are. An extensive discussion about the bounds of LOFs can be found in the original publication by Breunig et al. [44]. An artificial example of the LOFs for a set of points in 2D is presented in Figure 4.

The LOF of a tract is solely based on the number of k-NNs that will be used to assess its local tract density. Since, the value of 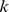 has a direct impact on the LOFs, Breunig et al. derived guidelines for reasonable values of 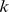 [44]. To achieve a stable solution without statistical fluctuations, it is sufficient to choose 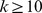. Beyond that, 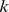 implicitly defines the minimum cluster size. Consider the smallest cluster 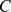 in a dataset with 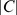 tracts. If 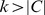, the k-NNs of each tract in cluster 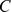 include not only tracts from the cluster 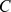 itself but also tracts that belong to another cluster or tracts that are outlier. In this case, LOFs will be artificially elevated. In order to prevent an unintentional increase of the LOFs for small clusters, the value 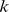 should not be higher than the number of tracts in the smallest cluster that is expected in the dataset: 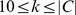.

#### Incorporating LOFs into the cluster analysis of fiber tracts

As LOFs specify a value for each tract that indicates how outlying a tract is compared to its k-most similar tracts, it is reasonable to incorporate this structural information into the clustering to support the outlier handling of CURE.

The outlierness information provided by the LOFs is used as complementary information besides the similarities to adjust the pairwise similarities between tracts during the formation of the clusters. Here, two scenarios have to be considered.

On the one hand, we have to consider intra-cluster distances. They are primarily related to the determination of the cluster representatives, which are iteratively selected by finding those tracts in the cluster that maximize the distance to all other tracts in the cluster. On the other hand, we also have to cope with inter-cluster distances that are used to determine those clusters that will be merged in each iteration of the clustering. In the latter case, the two clusters that minimize the distance between them have to be found (see Eq. 6). These two scenarios are obvious conflicting. While we try to maximize the distance between tracts in the first scenario, we try to minimize it in the second scenario. Consequently, both scenarios have to be handled differently (see below).

However, irrespective of the scenario, we first define a LOF-based correction factor 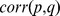 to adjust the pairwise similarities between two tracts 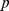 and 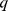. By denoting the local outlier factor for 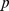, 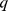 with 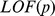 and 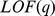, we define 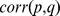 as follows:
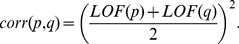
(7)


As long as 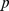, 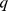 are not outlying and their LOF is around 1, 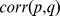 yields 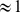. If either 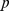, 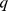 or both are outlying, the LOFs are elevated and the correction factor for 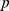 and 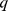 will increase by 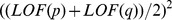.

#### Adjusting intra-cluster distances

Intra-cluster distances are used throughout the clustering to determine the cluster medoid and to select tracts of the cluster that act as representatives. However, this process promotes the selection of outlying tracts as representatives. As this might cause inferior clustering results, a careful selection of representatives is essential. We therefore adjust the intra-cluster distances to prevent outlying tracts from being selected, by using the LOF-adjusted distance 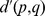 instead of 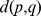 to assess the similarity between tracts 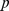, 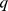:
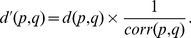
(8)


**Figure 5:**
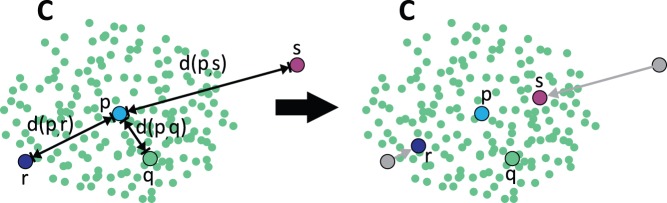
Influence of Local Outlier Factors on intra-cluster distances. Given one cluster 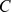 and the set of tracts 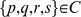, the influence of LOFs on the intra-cluster distances between 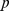 and the exemplary tracts 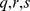 is unveiled. In the example, the LOF of 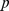, 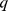 is approximately one, the LOF of 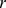 is slightly increased and the LOF of 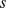 is considerably elevated. Since a reciprocal relation is used for the computation of intra-cluster distances compared to inter-cluster distances, high LOFs result in reduced distances between tracts – an attraction effect. Therefore, the LOF-corrected distance between 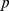, 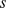 is considerably reduced, while the correction only slightly reduces the distance between 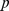, 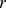. Since 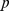, 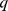 are not outlying (LOF 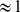), the LOF correction has almost no effect on the distance between 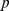, 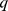.

The inverse application of the correction factor 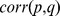 (Eq. 7) practically applies a spatially dependent attraction that pulls tracts with an increased LOF into the cluster. If tracts have a high LOF, the correction factor 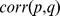 will be higher as well and the distance 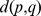 decreases. On the contrary, if tracts have a LOF that is 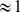, the tracts are practically not affected. Due to this reciprocal effect, tracts with a high LOF will suffer a penalty and have a reduced distance to the medoid and to other representatives (see Figure 5). This decreases the possibility that outlying tracts with a high LOF are selected as representatives of a cluster.

#### Adjusting inter-cluster distances

To adjust the distance between two different clusters 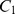 and 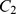 (see Eq. 6), we weight the distances 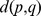 between two representative tracts 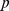, 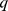 with the LOF-based correction 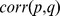. This yields the new LOF-adjusted distance, 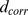, between 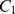 and 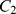:
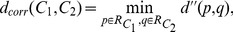
(9)

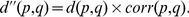
(10)


**Figure 6:**
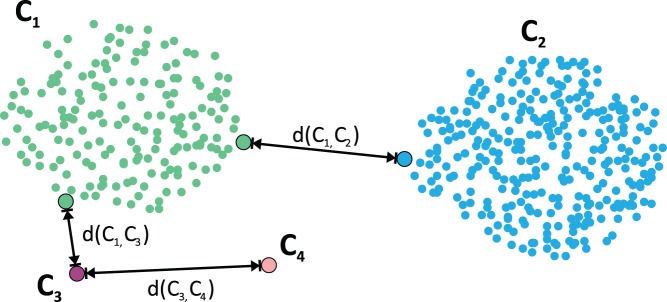
Influence of Local Outlier Factors on inter-cluster distances. Given clusters 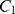
–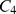 and the shortest distances 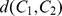, 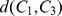, 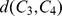 between the clusters, LOF-related effects on the inter-cluster distances are emphasized. Due to the cluster's surroundings, the LOFs for tracts in 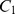 and 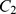 are approximately 1, whereas the LOF for tracts in the solitary clusters 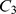, 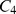 is increased and higher than 1. Hence, the LOF-based correction has almost no effect on the distance between clusters 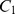, 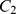. The distance between 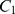 and 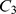 will be increased due to 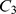
's high LOF. This repulsion effect is even more pronounced between clusters 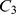 and 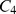, because tracts in both clusters have high LOF values.

This correction affects only the distances between the clusters and has an influence on when and which clusters are merged. In order to understand the mechanism behind the correction, the clustering should be considered as a continuous process in which clusters are not yet finished but are successively formed. If the LOF for the representatives of two distinct clusters is 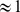, the distance between the clusters is not altered. By definition, these representatives have to belong to true clusters and are inside the clusters (otherwise the LOF would not be 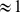). If representatives are located at the boundaries of a cluster (compare Figure 4), their LOF is slightly increased. As a result, the clusters will experience minimum repulsion and will be clustered slightly later due to their increased distance. If either one or both representatives of the clusters are outlier, they will possess a high LOF. As a result of the LOF-correction, the distance between the clusters will considerably increase and they will be merged substantially later (if they are merged at all). An exemplary illustration that depicts the adjustment of the inter-cluster distances for an artificial set of points in 2D is given in Figure 6.

This correction effectively contributes to the employed outlier elimination strategy, which is based on the assumption that outlying tracts will be far more isolated than other tracts. As a result of the LOF correction, outlying tracts or clusters will become even more isolated and will therefore be clustered not at all or in the very end.

#### Assigning and reassigning tracts

In CURE and CATSER, slowly growing clusters that contain only few tracts in the end of the clustering are labeled as outliers. Due to the randomized division of the data into multiple partitions during the clustering, tracts may be unintentionally labeled as outliers, even if a multitude of tracts are spatially located nearby in other partitions. In order to correct for possibly wrong outlier assignment, outlier are reevaluated. The distances to all extracted clusters are assessed and outliers are reassigned to the most similar cluster if the similarity between outlier and cluster is high enough.

**Figure 7:**
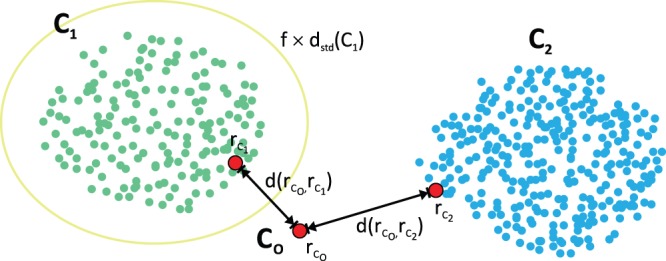
Assignment and reassignment of tracts in CATSER. Given the prototype clusters 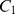, 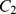, the solitary cluster 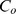 and the carefully selected subset of representatives 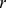, the closest cluster to 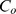 is 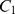 since 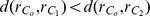. The representatives with the shortest distance are denoted as 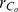 and 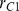. 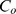 is then (re-) assigned to the closest cluster 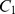 only if the distance 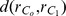 is smaller than 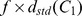, whilst 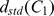 is the standard deviation of the distances between all representatives in 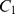.

By treating the outlying tract 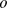 as a singular cluster 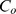 that consists only of tract 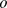, the distance 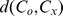 to all regular clusters 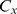 is computed, with 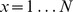 and 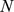 being the number of regular clusters. The cluster with the minimum distance to 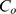 is denoted as 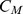 and the two closest representatives of 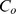 and 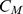 are termed 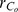 and 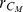 respectively. If 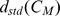 is the standard deviation of the distance between all representatives in 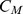 and the inequality.
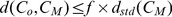
(11)


is satisfied, tract 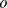 is assigned to 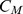 (see Figure 7). Otherwise 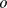 is labeled as outlier. The factor 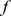 can be chosen arbitrarily and is used to regulate how similar tracts need to be, in order to permit the reassignment. A value of 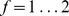 works usually quite well.

Subsequent to the clustering and the reassignment of wrongly labeled tracts, the set of tracts 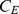 that were not part of the initial random sample (step 1 in Figure 2) have to be processed. Hereby, they are either assigned to the nearest cluster or labeled as outlier. This processing step is carried out in a similar way as the reassignment above. For each tract 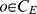 the distance to all regular clusters is computed and tract 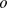 is assigned to the nearest cluster if inequality 11 is satisfied. As the computation of the LOFs for the entire dataset is too time consuming (the whole similarity matrix has to be available), we assume that the LOF of each tract in 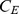 is 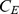.

### Integration of Anatomical Information into the Cluster Analysis

In order to incorporate anatomical information into the cluster analysis, we utilize a white matter atlas that contains various fiber bundles. As the clustering is based on the principle of merging clusters with the highest similarity, an effective way to influence the clustering is to modulate the distance 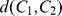 between the clusters 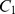 and 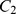:
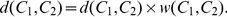
(12)


**Figure 8:**
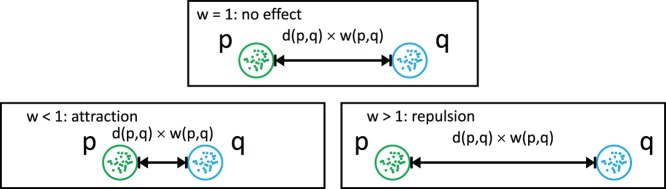
Effects of different weighting factors. The weighting factors guide the clustering by modulating the distance between the clusters 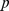 and 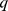 according to their anatomical correspondence in the atlas. While a weighting factor 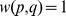 has no effect, a weighting factor 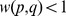 will decrease the cluster distance (attraction). Contrary, a weighting factor 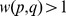 will increase the distance between the clusters (repulsion).

As those clusters with the shortest distance are merged in each step, the weighting factor 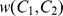 has a direct impact on the clustering and how clusters are formed. While values of 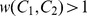 will increase the distances and lead to a repulsion, a weighting 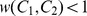 will result in attraction for 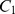, 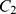 (see Figure 8). We exploit this effect and determine the weighting 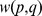 with respect to the atlas-class membership of the clusters 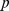 and 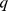. To guide the cluster analysis, this weighting is determined and applied in each successive step of the clustering as well as in the reassignment and the labeling stage. The steps in which the atlas has to be employed for the atlas integration are depicted in Figure 2 (steps 4 and 6–8), showing the atlas as optional input in grey dotted boxes.

#### The white matter atlas

For the cluster analysis, we use a voxel-based white matter atlas that was constructed with a semi-automatic method [25]. The atlas consists of various white matter (WM) structures (i.e. the classes of the atlas) and each WM structure contains a set of voxels that belong to the structure. Hereby, different regions can overlap and voxels are allowed to belong to multiple classes of the atlas. In Figure 1, a white matter atlas is shown with a selection of fiber bundles in distinct colors.

#### Determining the class membership for tracts

**Figure 9:**
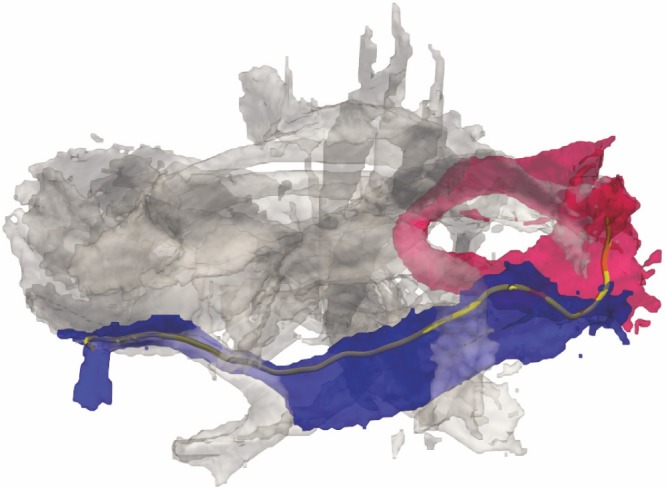
The figure shows a single fiber tract that traverses through two classes of an atlas. A single fiber tract (yellow) traverses through the inferior fronto-occipital fasciculus (IFO, in blue) and the forceps major (Fmaj, in red). The remaining atlas classes that share no spatial volume with the tract are displayed in grey. The class membership of the tract for the IFO is 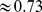 and for the Fmaj 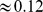.

In order to determine the weighting factor 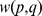, the atlas-class membership for the individual tracts and the clusters (groups of tracts) has to be assessed. The class-membership of single tracts is computed by rasterizing each tract and determining the spatial agreement of the tract and the classes in the atlas (Figure 9).

For each atlas class 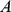 and each tract 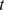, the spatial agreement 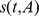 is computed using the number of all tract-voxels 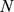 and the number of voxels 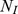 that intersect the class in the atlas.
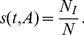
(13)


For a cluster 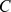 with a set of 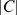 tracts, the atlas-class membership to class 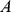 is computed by averaging the spatial agreement between all tracts in the cluster and class 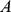:
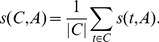
(14)


#### Determining the weighting factor for two clusters

During the clustering, the weighting has to be determined and applied in each successive step. For two clusters 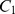 and 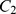, we identify the two classes of the atlas 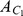, 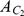 with the highest spatial agreement 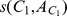, 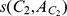. For improved readability, we will use the short notation 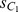 and 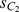 as abbreviation. Then, we distinguish between four cases:

- case 1: cluster 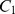 and 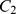 have no corresponding class in the atlas,
- case 2: either cluster 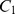 or cluster 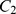 has a corresponding class in the atlas (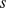 will be the spatial agreement for the non-empty class),
- case 3: both cluster 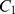, 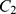 correspond to the same class in the atlas,
- case 4: both cluster 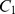, 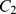 correspond to different classes in the atlas.

Each case modulates the weighting in a different way:
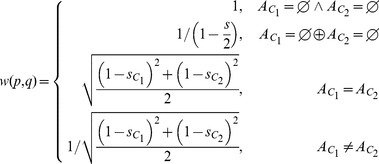
(15)


**Figure 10:**
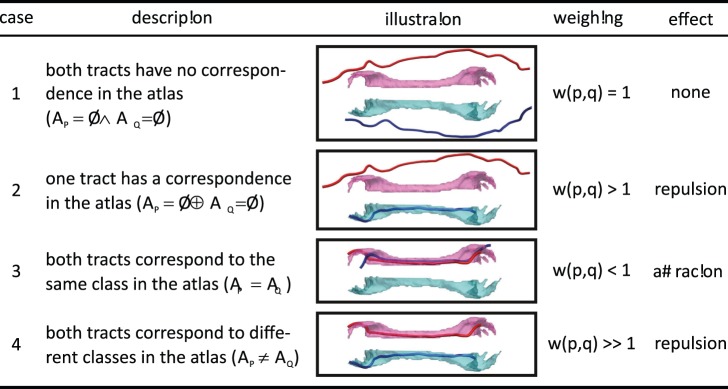
The four cases that determine the weighting factor for the atlas guidance. The figure shows the four cases that determine the weighting factor. In order to present and visualize the four cases, the classes of the atlas that correspond to the left and right cingulum bundle are shown in pink and cyan along with two tracts that represent two clusters (shown in red and blue).

The four cases are summarized in Figure 10. For the sake of simplicity, the clusters in the figure are shown as singular cluster with only one single tract. However, as clusters are merged together during the iterative clustering procedure, the cluster will grow in size and can thus be composed of multiple tracts. In this case, the spatial agreement between the cluster and the atlas classes is the average of the spatial agreement between all tracts and the atlas class (see Eqs. 13 and 14).

## Materials and Methods

### Data Acquisition and Data Processing

46 healthy subjects (24 female, 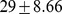 years old; 22 male, 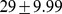 years old) were measured on a clinical 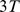 whole-body MR-Scanner (Tim Trio, Siemens Healthcare, Erlangen, Germany). This study was approved by the Ethics Committee of the Jena University Hospital. Participants provided informed written consent to participate in this study in accordance to the statement of the Ethics Committee.

For the diffusion tensor acquisition, a conventional single shot twice refocused Echo Planar Imaging (EPI) sequence was used with four bipolar diffusion gradients to compensate for eddy currents [45]. Fat suppression was achieved by applying a SPectral Attenuated Inversion Recovery (SPAIR) pulse. A 12 channel phased array matrix head coil was employed for the measurements. To minimize subject motion, special pads were used that secured a tight fit of the heads in the coil. The following acquisition parameters were used: a matrix of 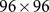, 55 slices with a thickness of 2.5 *mm*, voxel size 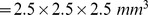, 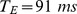, 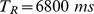, *α*  =  90° and acceleration factor 2. Six 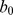 images without diffusion weighting (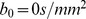) as well as 70 diffusion weighted images sampled with different gradient directions at 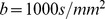 were acquired.

Following the data acquisition, in-plane interpolation was performed on the MR-Scanner, resulting in an effective voxel size of 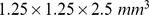. Due to the head fixation, subject motion was not observed and a retrospective motion correction was not performed. The Diffusion Toolkit [46] was used to perform DTI-based analysis and whole brain fiber tractography using the interpolated streamline method [6] with a fixed step-length of 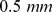 and an angle threshold of 41 degree. For the fiber tracking, three randomly located seed points were placed at subvoxel resolution in each voxel of the brain's white matter. The white matter seed mask was based on the FA maps with a manually-tuned minimum FA threshold of 0.2. Tracts having a length less than 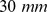 were subsequently removed from the dataset. On average, each dataset consisted of about 280 000 tracts with more than 20 million tract points per dataset. Tracts were not resampled and differed in both length and number of tract points.

All datasets were spatially normalized in a two step procedure, using the Advanced Normalization Tools (ANTs) [47]. In an initial step, a rigid transformation of the FA maps was applied to register and coarsely align all data to the common FA template (FMRIB58) of the FMRIB's Software Library (FSL) [48]. A subject-specific template was created by normalizing the datasets with the non-linear template generation framework of ANTs. As a starting point for this non-linear, spatial normalization process, the rigidly transformed FA maps were averaged to produce an initial FA template. The template was refined and improved in four iterations using the greedy SyN transformation model and the cross correlation metric of ANTs. The resulting transformation matrices and displacement fields were finally employed to transfer the fiber tracts into the space of the newly generated template.

### White Matter Atlas Generation

We constructed a white matter atlas with a semi-automatic method [25]. Out of the 46 spatially normalized datasets (see previous section), 15 randomly selected datasets were employed to generate the atlas. We selected 16 WM structures (WM bundles) to be included in the atlas: forceps major (Fmaj), the frontal projection of the corpus callosum (the forceps minor – Fmin) as well as the following bundles of the left and right hemisphere: anterior thalamic radiation (ATR), gyrus part of the cingulum cingulate (CGC), hippocampal part of the cingulum (CGH), cortico-spinal tract (CST), inferior fronto-occipital fasciculus (IFO), temporal part of the superior longitudinal fasciculus (SLFt), uncinate fasciculus (UNC). To delineate these bundles, a set of Regions Of Interests (ROIs) was drawn for each bundle, taking into account the guidelines for reproducible extraction by Wakana et al. [24]. For each dataset, tracts that crossed these ROIs were extracted and assigned to the corresponding WM bundle. While this is an efficient and fast way to extract the WM fiber bundles, it only extracts the major parts of the bundles. Tracts that belonged to the bundle but had not crossed all ROIs were not assigned to the bundle. This probably resulted in a loss of minuscule details for the bundles.

The probabilistic white matter atlas was then created by using the extracted bundles (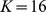) of all datasets (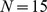). With all these bundles, the 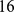 prototype classes of the white matter atlas were generated. Hereby, each class in the atlas contains all voxels that are associated with the corresponding atlas class and describes how reliably each voxel can be associated with this class. Let 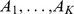 be the prototype classes. If 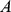 is one of these classes, it formally consists of a list of voxels 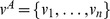 with an unknown number of voxels 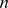 that belong to class 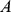. Each 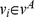 is a set of coordinates 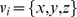 that describes the position of voxel 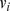 in the 3D dataset. The probability that voxel 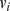 belongs to class 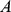 is denoted by 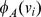. As 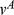 consists only of voxels that belong to 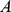, the probability for each voxel 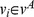 is 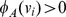. 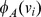 is therefore bounded by 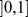.

To generate the probabilistic atlas a two step procedure was used. During the first step, the probabilities for each fiber bundle were computed individually for each dataset, before these probabilities were used to generate the final prototype classes in the second step.

For the first step, the computation of the dataset probability 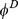 is performed individually for each dataset 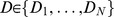. Initially, for each fiber bundle 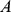 of dataset 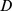, the tract density 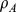 is determined. Hereby each tract that belongs to bundle 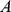 is rasterized to a user-defined 3D grid and all voxels 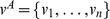 that are occupied by the tracts of 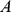 are identified. The tract density 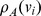 for voxel 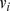 is computed by counting the number of tracts that occupy voxel 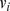. To obtain the dataset probability 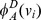 for voxel 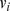 of bundle 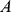, the ratio between the tract density 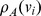 and the number of all tracts that occupy voxel 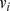 is computed:
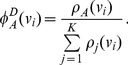
(16)


If only tracts of 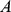 occupy voxel 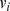, the probability 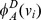 is 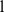.

After computing the dataset probability for each dataset and each fiber bundle the final prototype classes are generated in the second step. For prototype class 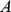, the prototype probability 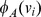 in voxel 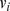 is defined as the average of all dataset probabilities 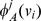 for 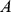 in voxel 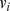 with 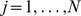:
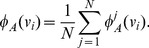
(17)


If there is no voxel in a bundle 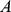 to which the corresponding bundles of all 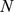 datasets contribute, the maximum bundle probability 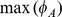 will be less than 1. To prevent such a degradation of probabilities, the probabilities in each bundle are normalized to a maximum probability of 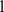.

**Figure 11:**
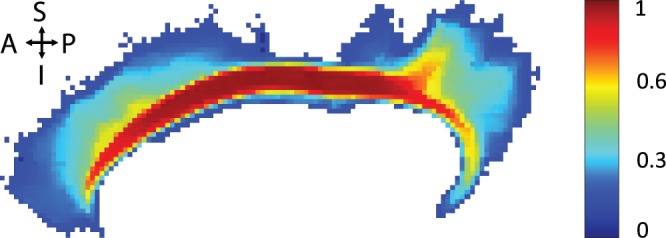
Example for an atlas class in the probabilistic white matter atlas. The maximum intensity projection of the probabilities is shown as a pseudo color image for the gyrus part of the left cingulum (CGC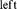). Regions with high probability (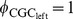) are colored in red, while regions with low probability (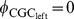) are shown in blue.

**Figure 12:**
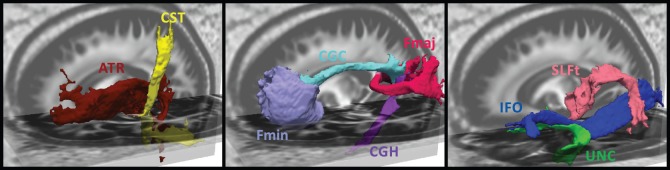
Volume renderings for a selection of fiber bundles defined in the white matter atlas. From left to right the bundles are: anterior thalamic radiation (ATR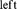), cortico-spinal tract (CST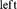), the forceps minor (Fmin), gyrus part of the right cingulum (CGC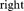), hippocampal part of the right cingulum (CGH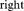), forceps major (Fmaj), inferior fronto-occipital fasciculus (IFO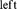), temporal part of the superior longitudinal fasciculus (SLFt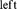), uncinate fasciculus (UNC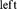).

During this prototype generation stage, tracts are rasterized to a 3D grid. As tracts are a set of real-valued points in 3D space, the atlas can be reconstructed for arbitrary grid resolutions. In this study we used an atlas with 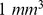 isotropic resolution. Unreliable voxels with probability less than 0.3 were removed from the atlas, before further processing was performed. An example for a class of the probabilistic atlas is shown in Figure 11. Volume renderings for a selection of fiber bundles that are defined in the atlas are shown in Figure 12. The bundles are overlaid onto the FA volume of a single subject.

### Spatial Matching of Clusters and Atlas Classes

After the clustering of a dataset, the obtained clusters are not ordered and a spatial matching is performed to relate the clusters to their best matching atlas class. For this purpose, the clusters of a dataset 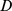 are rasterized to a 3D grid with the same resolution as the atlas. The tract density 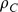 is computed for all clusters 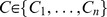 and normalized to a maximum density of 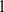 for each cluster.

The spatial agreement between all atlas classes and all obtained clusters is then determined. A matching value 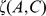 for an atlas class 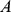 and a cluster 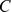 is computed that reflects the spatial resemblance between 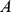 and 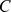 (see below). The higher the value 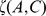 the higher the spatial agreement between 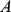 and 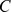. After the computation of the spatial matching value for all atlas classes and all obtained clusters, clusters are iteratively assigned to the best matching atlas class until all atlas classes have been associated with a cluster. During this process, a one to one mapping is enforced and clusters cannot be assigned to more than one atlas class.

To determine the matching value 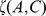, the intersecting voxels 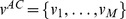 as well as the non-intersecting voxels 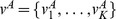 for 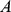 and 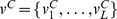 for 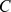 are identified. The number of all voxels in 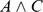 is denoted by 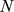. To assess the similarity in the overlapping regions of 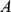 and 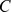, the average of the differences in the intersecting voxels is computed with:
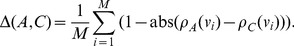
(18)


To take into account the average tract density of non-intersecting voxels for both 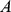 and 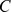, we also compute:
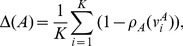
(19)

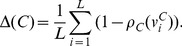
(20)


The matching value 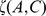 is then given by:
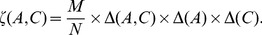
(21)


### Cluster Analysis

The clustering framework was implemented in C++ and uses highly specialized libraries as well as multithreading to accelerate the processing and reduce the computation time. Details and additional technical aspects of the implementation are discussed in the appendix (see section “Technical aspects and implementation details”).

For each of the 46 whole brain tractography datasets we performed the cluster analysis in the atlas-space with three different techniques: atlas-guided clustering with CATSER, conventional CATSER clustering (without atlas) as well as standard Hierarchical Agglomerative Clustering (HAC) using Ward's method [49]. For both atlas-guided and non-atlas-guided clustering with CATSER, we used the same parameters (see below). For HAC, the datasets were far to large to be processed in reasonable time. Therefore, we randomly selected 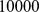 tracts from each dataset, clustered the sample individually and reconstructed 250 clusters for each dataset.

For the cluster analysis with CATSER, identical parameters, empirically derived from prior experiments were used. We chose a sampling size of 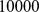 tracts (Figure 2, step 1), and divided the sample into 3 partitions (Figure 2, step 3) that were clustered during the preclustering stage (Figure 2, step 4). After 80% of the preclustering, outlier elimination was performed and very small clusters with less than three tracts were removed. During the final clustering stage (Figure 2, step 6) the second outlier elimination stage was performed after 60% of the clustering. This time, clusters with less than four tracts were removed. For the computation of the LOFs, the number of neighboring points 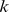 was set to 15. The number of representatives was set to a maximum value of 40 for a cluster size of 120 tracts. With respect to Eq. 5 this yields 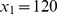 and 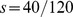. The factor 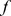 for reassignment of outliers was set to 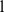 and for the labeling of unprocessed tracts to 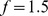 (see section “Assigning and reassigning tracts”). We reconstructed 250 fiber bundles for every dataset.

For the atlas-guided clustering with CATSER, the white matter atlas was used (see section “White matter atlas generation”). A subsequent step was employed, in which the spatial agreement of the clusters to all atlas bundles was assessed and a merging of clusters was performed if this merging would lead to an increased spatial agreement between the newly formed clusters and the best matching atlas regions. The purpose of this additional step is to guarantee that clusters are not splitted and completely formed.

The clustering of all datasets was performed twice with the three clustering techniques (see above). Each time a different distance measure was used to determine the similarity between the tracts. We used a Combined Distance (CD) measure [50] as well as the Hausdorff Distance (HD) (for details about the similarity measures we refer to section “Similarity measures” in the appendix). In total, cluster analysis was performed 276 times (46 datasets 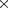 3 clustering techniques 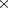 2 similarity measures).

With spatial matching (see section “Spatial matching of clusters and atlas classes”), clusters were identified and related to their corresponding and best matching class in the atlas. Inter-individual matching for bundles of different datasets was not performed, but can be applied in an additional spatial matching step, by selecting one dataset to which bundles of remaining datasets are matched. To evaluate the quality of the final results, we computed the spatial agreement (see Eq. 13) between the voxelized clusters and their corresponding, best matching atlas classes.

To demonstrate the benefits from outlier elimination, the effects of different outlier elimination strategies and varying levels of noise were investigated. For this purpose, one dataset that resided in its native space was segmented according to the guidelines by Wakana et al. [24]. The same 16 fiber bundles that are also defined in the atlas (see Figure 12) were extracted. For this segmented dataset, unsupervised clustering (without white matter atlas) was performed for varying levels of noise and different sets of outlier elimination parameters. As the correctness of fiber tract clusters are visually hard to depict due to their inherent complexity we used the Euclidean norm between the tract centroids as a similarity measure for this clustering experiment (see section “Similarity measures” in the appendix). Contrary to fiber tracts, the distance between the tract centroids can be easily depicted in 3D Euclidean space, which allows good visual delineation of the clusters and their shapes. For this experiment, the cluster analysis was performed for three different outlier elimination parameter sets (low, moderate and high outlier elimination, see Table 1 for details). In addition, artificial white noise was added to the tract centroids and gradually increased (0%, 33%, 66%, 99% additional noise).

**Table 1:** Parameters for the three different outlier elimination strategies.

Outlier elimination strategy	Preclustering		Final clustering	
	time point *t*_1_ in %	critical size *s*_1_	time point *t*_2_ in %	critical size *s*_2_
low	95	1	85	4
moderate	80	2	85	6
high	80	4	85	8

The table shows the outlier elimination parameters for the three outlier elimination strategies (low, moderate, high). The outlier elimination is performed during the preclustering (Figure 2, step 4) as well as during the final clustering (Figure 2, step 6). Clusters that contain no more tracts than the critical cluster size 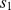 (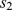) after 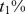 (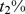) of the clustering has been finished are considered outliers and are removed from the subsequent clustering.

### Performance Analysis

In order to assess the performance of the clustering in a multiprocessing environment, a performance analysis was conducted using a symmetric multiprocessing (SMP) system equipped with 16 GB main memory and two Intel Xeon processors (L5430, quad core, 64-bit, 2.66 GHz). Effects on the execution time 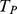 and the relative speedup 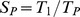 were investigated by gradually increasing the number of utilized cores 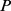. 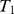 is the execution time if the processing is performed with a single CPU core. The performance analysis was conducted for the unsupervised clustering without white matter atlas. One dataset 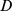 with 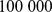 fiber tracts was used, whereas the reduced random sample consisted of 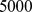 tracts. The Hausdorff Distance [29] and the Combined Distance [50] were used as similarity measures. To impede statistical fluctuations due to running background process, all computations were repeated ten times.

The analysis of our clustering framework is divided into three individual parts to identify those parts of the clustering (cmp. Figure 2) that are suspected to be computationally most critical, as well as to identify the parts that will profit the most from adding additional cores:

- Part 1: Computation of the similarity measures for 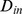 (Figure 2, step 2)
- Part 2: Clustering of the sample dataset 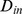 (Figure 2, steps 3–7):

The performance during the formation of clusters was investigated by gradually increasing the number of parallel clustered partitions.

- Part 3: Labeling of remaining tracts 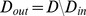 (Figure 2, step 8):

By employing identical clustering parameters as in part 2, the performance of the labeling was analyzed.

## Results

### Clustering

**Figure 13:**
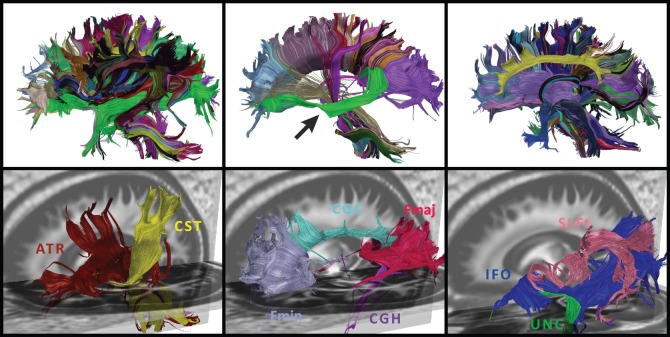
Atlas-guided clustering results for one dataset, clustered with the combined distance measure. In the top row all bundles are presented. Fiber bundles are shown for the left hemisphere (left image), bundles that cross the hemispheres (middle image) and bundles of the right hemisphere (right image). Different fiber bundles are displayed in distinct colors. In the middle image a tracking error is present that resulted in a fiber bundle connecting the prefrontal lobe and the corpus callosum (green bundle, marked with an arrow). The bottom row shows the clusters that correspond to the atlas classes that are shown in Figure 12.

The clustering of all 46 datasets was successfully performed using the three clustering techniques (atlas-guided CATSER, CATSER, HAC) and both similarity measures (CD, HD). For each clustering experiment and each dataset, we matched the clusters to the atlas classes and determined the best matching cluster for each class. Clusters for one exemplary dataset, processed with the atlas-guided CATSER clustering and the combined distance measure (CD) are shown in Figure 13. All extracted bundles of the dataset are displayed in the top row. To enhance the visualization, the bundles in the upper row have been divided into three groups: bundles of the left hemisphere (left image), bundles that cross both hemispheres (middle image) and bundles of the right hemisphere (right image). Fiber bundles are displayed in distinct colors, and tracts that belong to the same cluster are colored identically. The matched bundles that correspond to the atlas classes in Figure 12 are displayed in the bottom row of Figure 13 with the same coloring as in Figure 12.

**Figure 14:**
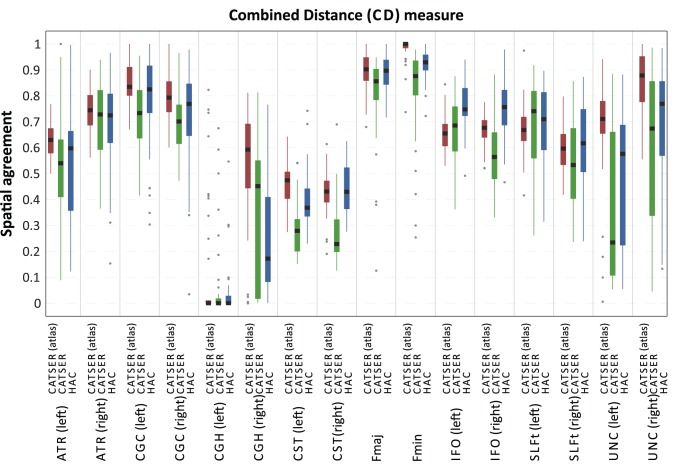
Spatial agreement of clustered fiber bundles and atlas classes for the combined distance measure. Using the atlas-matched fiber bundles of the 46 datasets that were clustered with the three different methods (atlas-guided CATSER, CATSER, HAC), we determined the average spatial agreement between fiber bundles and atlas class. The results for each bundle and each clustering technique are shown above.

**Figure 15:**
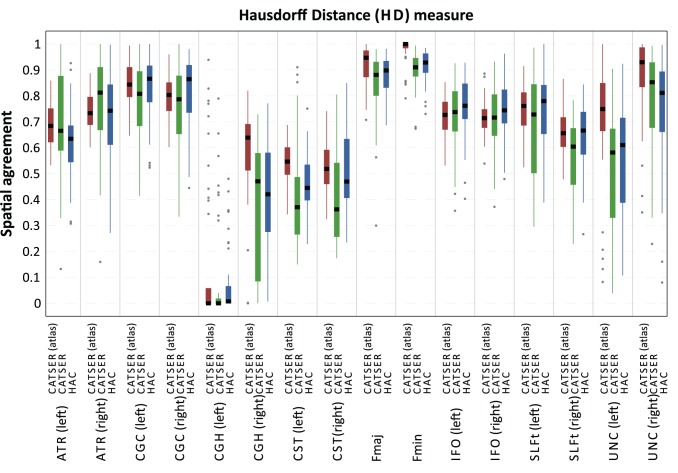
Spatial agreement of clustered fiber bundles and atlas classes for the Hausdorff distance measure. Using the atlas-matched fiber bundles of the 46 datasets that were clustered with the three different methods (atlas-guided CATSER, CATSER, HAC), we determined the average spatial agreement between fiber bundles and atlas class. The results for each bundle and each clustering technique are shown above.

To assess the quality of the clustering quantitatively, the spatial agreement (see Eq. 13) between matched bundles and atlas classes has been determined for all 46 clustered datasets. The spatial agreement for CD and HD is shown in Figures 14 and 15. Both figures use box plots to present the spatial agreement of the individual, clustered bundles, obtained with atlas-guided CATSER (in red), CATSER (in green) and HAC (in blue). The centerline in the boxes denotes the median (second quartile). The bottom and the top of a box correspond to the first and the third quartile and define the Inter-Quartile Range (IRQ). The lines that emerge from the top and bottom of a box are whiskers and contain all remaining data points that are in the range of 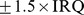. All data points that are outside of the whiskers are outliers and marked with grey dots.

For both similarity measures (CD, HD) all clustering techniques were able to group fibers into bundles that had a high spatial agreement with the classes in the atlas (see Figures 14 and 15). Nonetheless, for both measures various differences between the three clustering methods were observed. For all clustered bundles, the variability of the spatial agreement for atlas-guided CATSER clustering is considerably lower compared to clustering with CATSER and HAC. Especially the IRQs are significantly reduced, but also the number of outliers. By comparing the spatial agreement between CATSER (without atlas) and HAC no striking differences of the IRQs and outliers are apparent.

**Figure 16:**
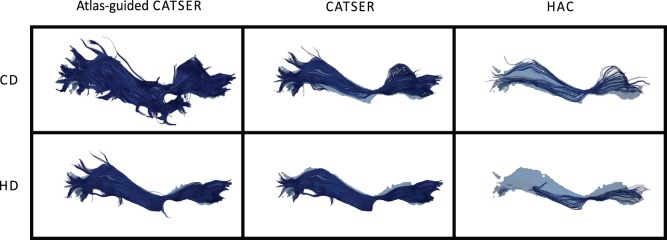
Inferior fronto-occipital fasciculus (IFO) of one dataset clustered with different methods. The IFO of one dataset is shown, which was clustered with all three methods (atlas-guided CATSER, CATSER, HAC) and both similarity measures (CD, HD). Bundles are shown in the atlas space and are superimposed with the corresponding class of the atlas (in semi-transparent blue). The spatial agreement for the bundles clustered using CD is (from left to right): 0.58; 0.62; 0.69 and for the bundles clustered using HD: 0.75; 0.67; 0.73.

By inspecting the average spatial agreement of atlas-guided CATSER and CATSER without atlas, we see higher spatial agreement for the atlas-guided clustering in most bundles (expect for the left IFO (for CD and HD), the left SLFt (for CD) and the right ATR (for HD)). In contrast, for the conventional clustering with HAC we observed higher spatial agreement in certain bundles than in corresponding bundles that were clustered with atlas-guided CATSER. However, it has to be considered that HAC clustered only a small subset of 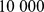 tracts, which resulted in considerably smaller clusters that occupied less space compared to the other techniques. As fewer tracts exist to delineate the whole bundle and to depict its smaller details, a higher spatial correspondence was obtained for HAC. This effect can easily be identified in Figure 16, which shows an example of a single fiber bundle (inferior fronto-occipital fasciculus – IFO), clustered with all methods. Using HAC, the best matching bundles contain only few fiber tracts. These few tracts travel primarily through the bundle, which results in a high spatial agreement. Regarding the other methods, the fiber bundle are more detailed and contain more fiber tracts. They resemble the atlas class more closer, even though the spatial agreement is lower compared to HAC (expect for the atlas-guided CATSER clustering with HD). For the atlas-guided CATSER with CD, however, the obtained cluster for the IFO is rather large and contains large parts of the uncinate fascicle compared to the clusters obtained with other methods. While the results for different clustering methods were consistent for both similarity measures, we observed that the spatial agreement with HD was consistently higher for most bundles compared to CD (Figure 14 vs. Figure 15). In average, the spatial agreement for HD compared to CD increased by 0.04 for atlas-guided CATSER, by 0.12 for CATSER and by 0.04 for HAC.

For the CGH (in particular the CGH of the left hemisphere) fiber bundles could not be extracted for all datasets. By analyzing the data we noted that no or only few fibers traversed through the volume of the CGH atlas classes for certain datasets. This is also captured in Figures 14 and 15, in which the CGH bundle has a very low spatial agreement and a high spread of values. This is presumably due to imperfections in the coregistration, the small volume of the CGH atlas class and the small number of tracts that this bundle contains. We assume that some of the very few tracts that belonged to the CGH were removed by the outlier elimination. The cortico-spinal tract also has a relatively low spatial agreement (Figures 14 and 15), which is the result of merging the CST bundle with tracts that are not part of the CST but are highly similar. While the CST is a very narrow fiber bundle, the tracts in its vicinity are still highly similar to the tracts that constitute the CST. Even though we incorporated anatomical information, some highly similar tracts are still merged into the CST, which leads to a reduction in spatial agreement (compare the occupied volume of the CST atlas class and the corresponding CST cluster in Figures 12 and 13).

**Figure 17:**
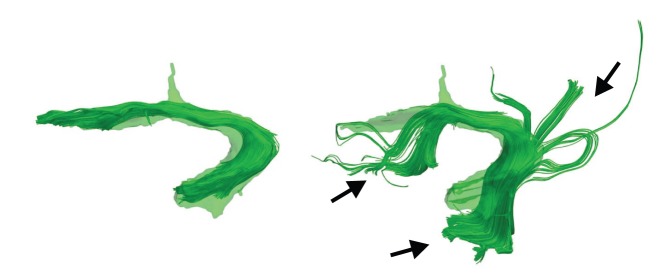
Left uncinate fasciculus (UNC) of two datasets clustered with atlas-guided CATSER clustering. Both bundles reside in the atlas space and are superimposed with the corresponding class of the atlas (in semi-transparent green). While the bundle on the left follow the anticipated trajectory of the UNC, the bundle on the right side contains additional tracts that leave the bundle and follow other paths. The spatial agreement for the left bundle is s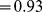 and for the right bundle s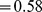.

With the atlas-guided CATSER cluster we observed an elevated IRQ of the spatial agreement for certain bundles (e.g. UNC). This increase is primarily associated with the inclusion of additional tracts during clustering that belong only partly to the bundle. In Figure 17 one example is presented that shows the uncinate fasciculus (UNC) of the left hemisphere for two datasets and the volume of the corresponding atlas class (in semi-transparent green). While the tracts follow the anticipated trajectory of the fibers in the clustered UNC in the left image, the bundle in the right image contains additional fibers that leave the bundle (arrows). These incorrect tracts share major parts of the atlas-class volume and also have a high partial correspondence to other tracts in the bundle.

**Figure 18:**
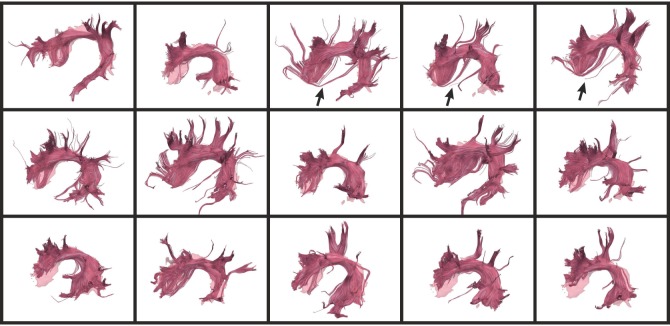
Temporal part of the left superior longitudinal fasciculus (SLFt) for 15 datasets. The datasets reside in the atlas space and were clustered with the atlas-guided CATSER clustering. The spatial agreement for the bundles in the top row is (from left to right): 0.62; 0.65; 0.67; 0.61; 0.68, in the middle row: 0.67; 0.60; 0.82; 0.64; 0.60 and in the bottom row: 0.64; 0.61; 0.74; 0.65; 0.69. In certain images, tracking errors have been observed that connect the SLFt to the external capsule (see arrows).

In order to demonstrate the consistency of the atlas-guided CATSER clustering, Figure 18 displays the temporal part of the superior longitudinal fasciculus (SLFt) for different datasets. The clusters are superimposed on the volume of the corresponding atlas class. Even though clusters vary between subjects, the appearance, the shape and size of the cluster is very similar and close to the volume-based atlas representation. Due to the fact that various sub-parts of the SLFt leave the atlas class, the spatial agreement is decreased and ranges between 0.60 and 0.82 for the shown bundles.

During the analysis of the clustered datasets, some tracking errors were observed. In Figure 13, for example, various tracts were traced by the tracking algorithm that connect the prefrontal lobe and the corpus callosum (green bundle in the middle image of the top row, marked with an arrow). Another tracking error is shown in Figure 18, in which the SLFt bundle of various datasets connect to the external capsule in the sub-insular white matter (see regions of the bundles marked with an arrow).

### Outlier Elimination

**Figure 19:**
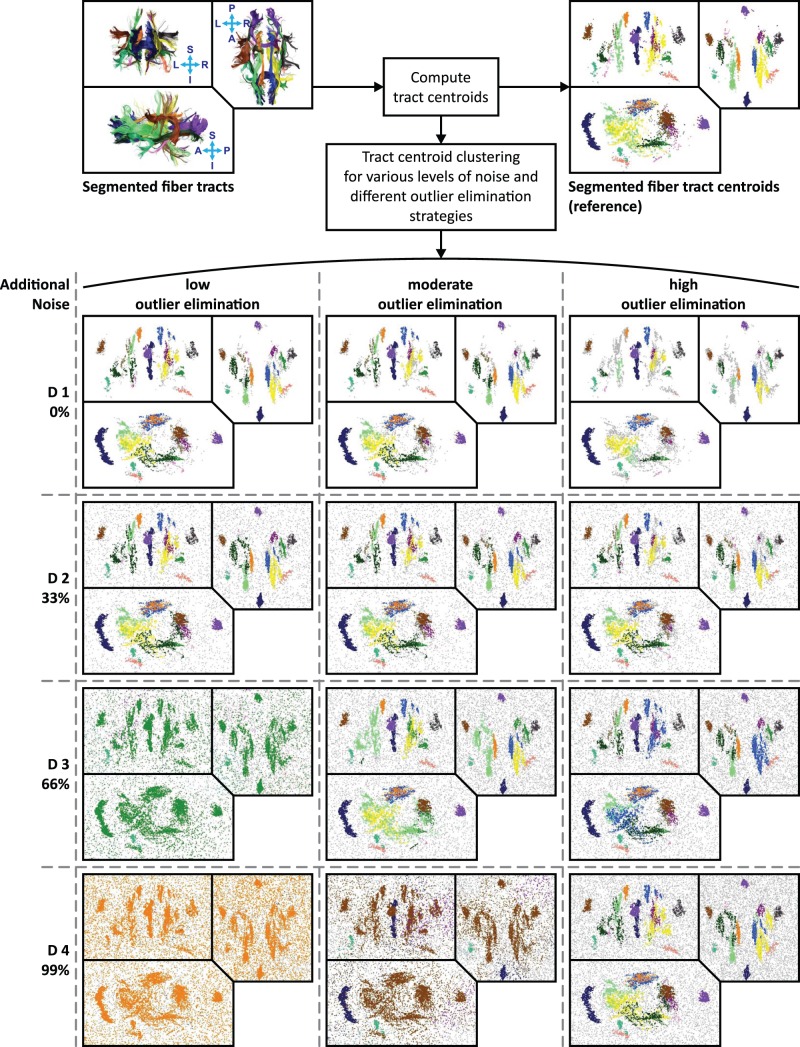
Results of the experiments that were conducted to demonstrate the effects of outlier elimination. One dataset was segmented into a set of bundles that are shown in distinct colors in the top left corner. Using the segmented fiber tracts, centroids of tracts were computed and used as a gold standard. Each point in the gold standard image (top right) represents the centroid of one fiber tract. The groups of centroids (gold standard) are show in the same color as their corresponding fiber bundles. All data is presented in a tableau view that shows the data in three different orientations: top left image = anterior-posterior view, bottom left image = left-right view, top right image = superior-inferior view. The centroids of the gold standard dataset were clustered using the tract centroid distance as similarity measure. Varying levels of artificial noise (0%, 33%, 66% and 99% additional noise) were used and different outlier elimination strategies were tested. The results for the clustering are displayed in the 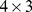 table (D1–D4).

Results of the outlier elimination are presented in Figure 19. The fiber tracking dataset in the upper left corner was manually segmented and the 16 fiber bundles that are also defined in the atlas were extracted. The data was not spatially transformed and resides in its native space. For visualization the data, a tableau view shows the data in three different orientations: top left image = anterior-posterior view, bottom left image = left-right view, top right image = superior-inferior view. Each of the segmented fiber bundles is shown in a different color. For all tracts of the dataset the centroids were computed and used as gold standard. Centroids are shown in the top right corner, with the same tableau view and identical coloring as the tracts.

To investigate the clustering with CATSER, the centroids were clustered with different outlier elimination strategies (low, moderate, high outlier elimination) and varying levels of noise (D1 = 0%, D2 = 33%, D3 = 66%, D4 = 99%). The results for this tract-centroid clustering are shown below the gold standard datasets (tracts, centroid). Again, the tableau view was used to visualize the results of the clustering from three distinct orientations. Resulting clusters are shown in different colors, while the colors of the clusters were selected in correspondence to the coloring of the gold standard dataset (top right tableau in Figure 19). Centroids that were identified as outlier during the clustering are displayed in grey.

First of all, we observed that the transformation from the segmented tracts (top left) to centroids (top right) already resulted in some isolated centroids that are visible in the tableau of the (segmented) fiber tract centroids (the gold standard).

By inspecting the clustered tableaus in Figure 19, it is evident that the quality of the clustering depends on both the level of noise in the dataset and the outlier elimination strategy. Overall, we observed that the framework was able to handle all scenarios quite well. However, no outlier elimination strategy was able to achieve an adequate clustering for all four noise scenarios (D1 D4). If noise is barely present (D1, D2), minimal outlier elimination is sufficient to obtain a good discrimination between the clusters, while a more aggressive outlier elimination strategy is indispensable if too much noise is present (D3, D4). If more aggressive outlier elimination strategies are used for datasets with little noise (e.g. D1, D2), the outlier elimination rate might be too high and small clusters might be labeled as outliers. In the opposite case, when plenty of noise is present (e.g. D3, D4) and minimal outlier elimination is used, clustering will probably fail and lead to erroneous clusters. In general, the number of points that were identified as outliers increases with intensified outlier elimination. Hence we believe that a moderate outlier elimination strategy seems to be a good compromise.

However, an increase in noise always has harmful effects on clustering and will result in degraded performance due to noise contaminations of the true clusters. The clustering methods will have difficulties to identify the correct clusters and chaining effects will lead to merging of distinct clusters. The more noise the more aggressive the outlier elimination should be in order to achieve satisfying clustering with good delineation of fiber bundles.

### Performance Analysis

**Figure 20:**
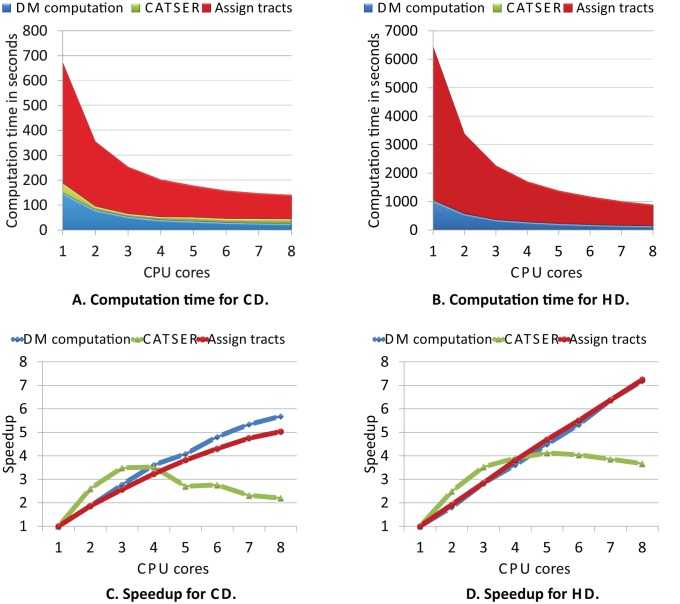
Performance analysis of the cluster analysis for the similarity measures CD (left) and HD (right). The overall computation time is shown in the top row and the achieved speedup below. For the analysis, the whole clustering process was separated into three distinct parts and analyzed separately (see section “Performance analysis” in Materials and methods). In all plots, each part is highlighted in distinct colors, whereas blue denotes the computation of the distance matrix (part 1: “DM computation”), green the clustering (part 2: “CATSER”) and red the labeling of remaining tracts (part 3: “Assign tracts”). For both distance measures, we observed that part 1 and part 3 were the most time consuming stages during the clustering. By utilizing multiple CPUs during both parts, a high speedup was achieved and the computation time was significantly reduced. While the speedup is nearly optimal for HD, the speedup for CD is slightly reduced. However, the overall processing speed for CD is still 10 times faster.

The performance analysis of our clustering framework was conducted as described in the Materials and methods section. Figure 20 shows the results of the analysis for the individual parts of the cluster analysis and the two analyzed similarity measures (CD, HD). The overall computation time in dependency of the number of employed CPU cores is shown with a stack plot in the top row, along with the corresponding speedup in the bottom row.

Due to the separation of the similarity measures from the clustering, precomputation of the similarity measures (part 1, step 2 in Figure 2) and assignment of remaining tracts to the prototype clusters (part 3, step 8 in Figure 2) were the most time consuming parts that accounted for major portions of the computation time. The actual clustering process that is responsible for the generation of prototype clusters from the sample (part 2, steps 3–7 in Figure 2) accounted only for a fraction of the total computation time.

Increasing the number of active CPU cores led to a significant reduction of the computation time for both distance measures. For part 1 and part 3, the speedup is nearly linear for CD (Figures 20C), whereas the speedup is fully sustained for HD as the number of employed cores increases from 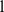 to 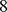 (Figure 20D). This implies that the HD could be parallelized very efficiently, whereas the additional CPU cores are not as efficiently used with CD. However, cluster analysis with CD is still an order of magnitude faster compared to cluster analysis with HD (Figure 20A and B).

While the performance analysis was only performed for unsupervised clustering, we observed an increase in computation time of approximately 10% for atlas-guided clustering.

## Discussion and Conclusion

Due to the high computational complexity of many conventional clustering algorithms, cluster analysis is in practice restricted to small datasets (e.g. [27], [29], [33]). In this contribution, we have introduced a new framework for cluster analysis of tractography datasets derived from diffusion-weighted MRI data. To assess the similarity of fiber tracts, the framework contains several proximity measures that can either be used independently or in conjunction with other similarity measures to perform clustering in higher dimensions. Together with practical concepts for cluster analysis of large tractography datasets, the new clustering method CATSER has been presented. CATSER exploits the inherent redundancy of large datasets and separates fiber tracts into meaningful bundles in reasonable time. Structural information of a white matter atlas can be incorporated into the clustering to achieve an anatomically correct and reproducible grouping of fiber tracts. This approach combines the benefits of classification and clustering and can be considered a hybrid technique. In addition to the clusters that correspond to the classes of the atlas, CATSER also extracts additional clusters that are concealed within the dataset. If an atlas is not available (e.g., in pediatric cases [51]), the technique can still be used to cluster large datasets without anatomical guidance. 46 tractography datasets of healthy volunteers have been processed and clustered with three different clustering techniques and two similarity measures (Combined Distance (CD) and Hausdorff Distance (HD)). The presented clustering technique CATSER (with and without atlas-guidance) and conventional Hierarchical Agglomerative Clustering (HAC) was used for clustering. With HAC only a small subset of 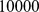 tracts was clustered due to the high computational complexity of HAC.

For the majority of the fiber bundles that corresponded to classes in the atlas, consistent results were obtained. In order to quantify the quality of the clustering the spatial agreement between clustered fiber bundles and their corresponding atlas classes was assessed. For both similarity measures we observed that the incorporation of the volumetric atlas clearly resulted in an improved, more consistent and reproducible clustering of fiber tracts. While spatial resemblance to the atlas classes increased, variability decreased. In contrast, both other methods (CATSER without atlas and HAC) had significantly higher spread of values for spatial agreement.

The comparison of the utilized similarity measures revealed that spatial agreement was consistently higher for HD compared to CD. Even though we expected that incorporating shape similarity would improve clustering and increase spatial agreement between clusters and atlas classes, this assumption was not confirmed. One reason for the lower spatial agreement may be related to the shape similarity which is an essential part of the CD. The shape similarity is not computed for the entire tracts but only for matched partial tract parts to determine the similarity of complete and incomplete tracts (see section “Similarity measures”). If the shape of the matched tract parts is highly similar, agreement rates are also high even if the tracts are not incomplete but belong to another fiber bundle. Figure 16 contains an example where the IFO contains a substantial part that belongs to the UNC. Nevertheless, one has not only to consider spatial agreement but also computation time. Here, the cluster analysis with CD was 10 times faster compared to the cluster analysis with HD.

The spatial correspondence between obtained clusters and atlas classes was computed by using the number of all cluster voxels and the number of cluster voxels that intersect the atlas class (see Eq. 13). While this takes into account voxels that are occupied by both – the cluster and the atlas class, it entirely neglects the possibility, whether tracts have been falsely assigned to the fiber bundle and occupy voxels that belong to other atlas classes. To quantify the correctness of the obtained clusters more accurately, future studies should ideally use measures that consider not only voxels that occupy the corresponding atlas class but also voxels of the cluster that are situated in different classes.

The separation of the similarity computations from the clustering permits the presented framework to dramatically reduce the computation time of the cluster analysis by utilizing multiple CPUs of modern multiprocessor systems. We analyzed the performance of the clustering framework and demonstrated the good speedup of the framework in a multiprocessing environment. The framework delivers nearly optimal speedup and performance, as long as the employed similarity measures are implemented efficiently. If a conventional fiber tracking dataset is available together with a white matter atlas, the presented technique CATSER can be used to consistently and reproducibly extract fiber bundles that correspond to the regions of the atlas. Especially in the light of the recent introduction of algorithms for generating and visualizing fiber tracts with the MRI scanner software of vendors, methods and tools for fast and correct extraction of fiber bundles are becoming increasingly important in order to aid the medical personal in analyzing and interpreting tractography data. Besides the application of CATSER for clustering such single subject datasets in clinical routine, we believe that the presented techniques and algorithms are especially useful and important for the fast and fully automated clustering of multiple datasets that are supposed to be analyzed with fiber bundle driven techniques for quantitative analyses.

As an anatomically correct and consistent grouping of tracts for multiple subjects is difficult to achieve without additional anatomical information, our technique can incorporate an atlas to guide the clustering process. We demonstrated that CATSER is capable to extract bundles that are defined in the atlas and that it also extracts additional reasonable clusters with conventional clustering. To influence the cluster generation we computed the spatial correspondence between tracts and atlas classes and incorporated this information into the clustering. As the atlas contains not only the occupied voxels for each atlas class but also additional probabilistic information, it might be reasonable to incorporate this information in the future. The atlas contains only the major parts of the bundles due to the employed ROI-based extraction technique. Even though the spatial agreement between obtained clusters and atlas classes was already quite high, we anticipate that the manual generation of an atlas that captures even smallest details will increase the spatial agreement between the clusters and the atlas bundles even further.

The validity and anatomical correctness of the obtained clusters is not only affected by the cluster analysis algorithm. Both data quality and preprocessing techniques play a crucial role and influence the generation of the white matter atlas and the cluster analysis. In this study, we only used Diffusion Tensor Imaging (DTI) and deterministic tractography as such techniques are widely available and often used in clinical routine [51]. However, the measured diffusion signal is just an approximation of the underlying microstructure that consists of billions of axonal connections per imaging voxel [52]. Even though the diffusion tensor is a good approximation if tissues are homogeneous and axons are aligned parallel, it is insufficient in heterogeneous tissue, where multiple fiber compartments give rise to a complex diffusion signal (e.g. crossing, fanning or kissing fibers). As the tensor degrades, DTI is incapable to describe the microstructure properly. In such cases, tractography often fails to reconstruct meaningful fiber tracts. Tracts may be incorrect, will be disrupted along the course of the structure or even perform an unreliable trajectory that follows distinct anatomical structures nearby. Examples for tracking artifacts and tracts that connect wrong regions are seen in Figures 13 and 18. A cluster that consisted of additional spurious tracts is shown in Figure 17. While the clustering technique can counter some of these imperfections by using outlier elimination, it will be unable to identify incorrect tracts if a sufficiently large number of similar misguided tracts exist. If tracts share major parts of the atlas-class volume and also have a high (partial) correspondence to the other tracts in the bundle, the atlas-guided clustering is limited in excluding incorrect fibers. In order to describe the diffusion in the presence of complex fiber architectures accurately more elaborate methods have to be used. With complex techniques that sample the diffusion signal in many directions with high b-values (e.g. high angular diffusion imaging [53], [54]), Orientation Distribution Functions (ODFs) can be reconstructed that characterize the diffusion signal more accurately and are able to resolve regions with crossing fibers. By employing high angular resolution diffusion imaging in conjunction with ODF-based tractography techniques [55], we anticipate significant improvements in the quality of the tractography datasets and a reduction of unreasonable clusters. As the white matter atlas is generated on the basis of reconstructed fiber tracts, erroneous tractography influences the atlas generation as well. The connection between the uncinate fasciculus and the inferior frontal gyrus (see Figure 17) is, for example, missing from the atlas. Considering heterogeneous tissue where multiple fiber compartments exist (e.g. crossing fibers), a voxel-based probabilistic atlas is limited as probabilities will be significantly reduced in regions with crossing fibers. An orientation dependent atlas that takes into account the fiber orientation in each voxel by using, for example, ODFs, will be the next reasonable step to further enhance the clustering and to prevent the bundling of misguided tracts. In addition, a grey matter atlas might also be employed to improve the clustering. In this case, a weighting factor might be based on the distance between the tracts and their closest grey matter region.

Our atlas-guided clustering approach aims at a fast and consistent extraction of fiber bundles for multiple subjects by using a white matter atlas. Compared to recent techniques that have been presented (e.g. [35], [39], [40]), it enables the anatomically correct extraction of bundles that are defined in the atlas, as well as the extraction of additional bundles that are not available in the atlas. While Guevara et al. [40] uses a reasonably fast approach that is based on the clustering of voxels, Visser's algorithm employs partitioning and repeated hierarchical clustering to achieve a grouping of tracts. Even though Visser's technique [39] works quite well for the clustering of fiber tracts, it is limited by the long computation times, which make it impractical to cluster large numbers of datasets. However, while such similarity-based approaches can be used for grouping fiber tracts, a good correspondence of the clustered bundles to the true anatomical bundles cannot be guaranteed due to the lack of anatomical knowledge. As a result, Guevara et al. extended their voxel clustering approach to increase anatomical correspondence by incorporating a white matter atlas [56]. First, agglomerative voxel clustering is performed until many thousands of clusters remain. This multitude of clusters is then classified using a manually labeled white matter atlas, derived from a set of clustered datasets. A different approach for the anatomically correct extraction of white matter fiber bundles was proposed by Yendiki et al. [57] with the TRACULA framework. Instead of using cluster analysis to retrospectively bundle fiber tracts from whole brain tractography datasets, TRACULA aims at the separated reconstruction of the bundles during the fiber tracking itself. Hereby, probabilistic tractography is used to reconstruct only the tracts of interest by restricting the tracking using anatomically a-priori-defined regions of interest as defined by Wakana et al. [24].

Cluster analysis of fiber tracts is not only based on the clustering algorithm itself, but fundamentally depends on the employed similarity measures that define the criteria to distinguish fiber tracts. Popular measures such as the Hausdorff distance [29], the Chamfer distance [58] or shape-based similarity measures [59] are often used to assess the similarity of entire tracts, but neglect partially overlapping [60] or incomplete fibers. To take into account partially overlapping fiber tracts, Wassermann et al. [60] proposed a mathematical framework that modeled tracts and fiber bundles as Gaussian processes using blurred indicator functions in voxel space. While the Combined Distance measure (CD) also takes into account incomplete or partially overlapping fibers, Wasserman's similarity measure has also some resemblance to our atlas-guided clustering. In their framework tracts are successively formed and the Gaussian processes of the tracts are merged in each iteration. As this approach is partly similar to our atlas-guidance, both methods might by suitable to complement each other.

Finally, the scalability of our clustering framework was analyzed with respect to the number of employed CPU cores. Results revealed that the computationally most demanding parts are the computations of fiber tract similarities, while clustering with CATSER itself is quite fast. Thus, the computation time is primarily determined by similarity computations. An in-deep analysis demonstrated that the clustering framework enables fast processing with minimal computational overhead. Even when all cores of a system are employed, the achieved speedup is nearly optimal. However, results indicate that speedup of the framework depend on the utilized similarity measure. This was confirmed by additional experiments, which revealed that a reduced speedup for certain similarity measures is primarily related to the computer system's memory management. We assume that the degraded multiprocessing efficiency is the result of a memory bandwidth bottleneck because of the system's inability to handle excessive memory (de-)allocations that may occur during the computation of certain similarity measures. Although the presented framework already offers good performance, an additional gain may be achieved by developing more sophisticated algorithms for determining fiber tract similarity or by exploiting parallelism with alternative hardware architectures. Especially General Purpose Graphics Processing Units (GP-GPUs) that are based on a stream processing architecture [61] already demonstrated their potential to reduce the processing time for cluster analysis of fiber tracts notably [62].

## Similarity Measures

### Tract Centroid Distance Measure (TCD)

We define the tract centroid distance 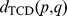 between two tracts 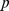, 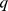 as the Euclidean distance between their centroids 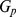 and 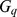:
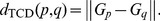
(22)


For an arbitrary tract 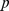 described by a finite set of tract points 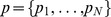 and 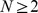, the tract centroid 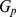 is 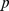
's center of gravity. This is necessary to cope with unequally distributed points along 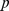 that would shift the centroid. Two successive points 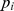 and 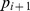 define an individual segment of 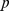. The centroid and the length for each segment is then denoted by 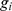 and 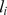, respectively:
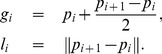
(23)


The center of gravity for 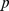 can now be defined as the length-weighted average of all segment centroids of 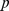. If 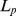 is 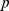
's overall tract length:
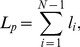
(24)



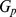
 is given by.
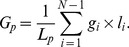
(25)


### Tract Orientation Similarity Measure (TOS)

In order to determine the tract orientation similarity 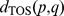 between two tracts 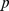, 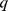 with 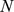, 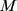 tract points, we compute the angular distance between their orientation vectors 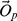 and 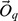:
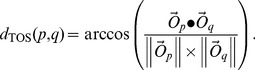
(26)


As orientation vector 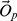, 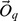 we use the endpoint-orientation of both tracts [46]:
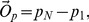
(27)

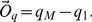
(28)


### Hausdorff Distance Measure (HD)

For two tracts 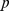 and 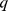 with a finite set of tract points 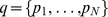, 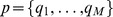 and 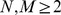, the one-sided Hausdorff distance is defined as the maximum of the shortest distances between the points of 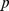 and 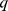:
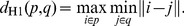
(29)


Since the Hausdorff distance is directional (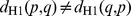), we define the two-sided Hausdorff Distance (HD) as follows:
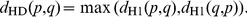
(30)


### Combined Distance Measure (CD)

The Combined Distance (CD) measure [50] is based on the assumption that the spatial location, the orientation and the shape of the tract are the most important characteristics of fiber tracts. Consequently, the CD uses these properties to determine the similarity between two tracts 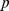, 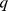 with 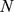, 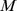 tract points. To determine the spatial location and the orientation of the tracts, we use the tract centroid distance 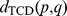 and the tract orientation similarity 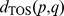 (see definition above). Assessing the shape similarity between tracts is more complex. For this purpose a method of Atalla et al. [63] based on tract resampling, contour segmentation and elastic matching was adopted [50]. To cope with incomplete tracts, we use the resampled tracts to determine the two points that minimize the distance between 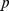 and 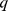. Using these two points a partial tract matching is performed and the sub-tracts 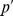 and 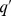 are extracted. For these sub-tracts the tract centroid distance 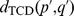 and the tract shape similarity 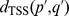 is computed (cmp. [50], [63]).

Even though these measures can be employed individually or combined for multidimensional cluster analysis, we decided to merge the individual measure into one measure. The one-sided combined similarity measure 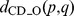 is then defined as the sum of the individual, normalized (between 0 and 1) measures:
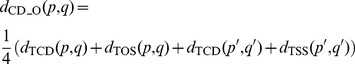
(31)


At this point, it might be also beneficial to use individual weights to favor or to penalize the individual measures [64]. To account for reversed tracts the procedure is repeated for the reversed tract 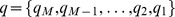 which yields 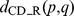. The similarity between tract 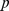 and 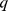 is then the minimum of 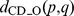 and 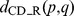:
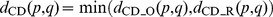
(32)


## Technical Aspects and Implementation Details

To facilitate fast processing of large datasets the framework was implemented in C++ for 64-bit Linux (x86–64) architecture. Various software packages, such as the Boost C++ Libraries [65], the Automatically Tuned Linear Algebra Software (ATLAS) and the AMD Core Math Library (ACML) were employed. In addition to these specialized libraries, the framework heavily relies on Symmetric MultiProcessing (SMP) architectures with multiple compute cores and a shared memory model. By these means multithreading with 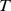 threads is employed to further reduce computation time and to accelerate the processing during stages that are computationally demanding.

For a dataset 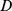 with 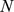 tracts three basic processing stages exist:

1. Extraction of a random sample of tracts 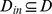 with size 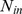.
2. Performing the cluster analysis of 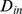 to obtain a set of prototype clusters 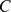 with size 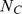.
3. Assigning remaining tracts 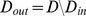 to 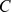 with size 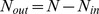.

**Figure 21:**
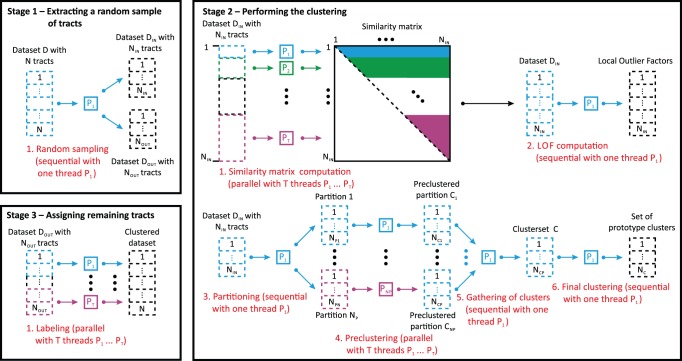
Overview of the three fundamental stages of the clustering and their sequential and parallel sub-stages. The figure depicts the way how the data is processed during the stages of the clustering process. It is illustrated, which parts of the clustering are performed either in serial or in parallel and how the data is distributed across multiple threads.

Reduction of the processing time is accomplished by analyzing stages and sub-stages of the cluster analysis first. Computationally demanding parts are optimized and parallelized. An overview of both, the sequential and the parallel stages in the framework is given in Fig. 21.

### Stage 1– Extracting a Random Sample of Tracts

Finding a random sample 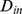 of tracts in a dataset 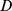 is computationally not demanding and can be carried out serially. As the Fisher–Yates shuffle [66], [67] guarantees unbiased results, it is employed for the extraction of the reduced random sample.

### Stage 2– Performing Clustering

Performing the cluster analysis for the reduced set of tracts in 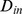 consists of several sub-stages – the precalculation of the local outlier factors, the partitioning of the data, the preclustering and the final clustering that produces 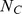 prototype clusters.

The computationally most expensive operation is the recurrent calculation of the pair-wise similarities between all tracts in 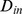 that is necessary in all stages but the partitioning stage. Consequently, similarities are computed only once and stored on disk before LOFs are calculated.

Since the entirety of similarities must be available, 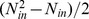 similarities are computed for every feature to obtain the triangular 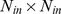 similarity matrix 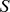. This, however, does not cover the computational complexity necessary to compute the pair-wise similarity for two tracts. Employing complex features (e.g., Hausdorff distance) will increase the computational complexity further.

To reduce the computation time, the calculation of the triangular matrix 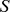 is sourced out to multiple threads and performed in parallel. Matrix 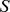 is divided into an arbitrary number of contiguous line-segments, with an approximately equal number of tracts (since the matrix is triangular, the number of elements differ from line to line). All similarities related to one tract are calculated by one thread.

LOFs are then computed by employing the precalculated similarities.

For the preclustering, the Fisher–Yates shuffle is employed to shuffle the tracts in 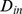, before 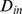 is partitioned into 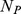 sets 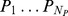 with approximately equal size 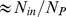. The partitions are then clustered simultaneously. Later on, the final clustering is carried out serially.

### Stage 3– Assigning Remaining Tracts

Since the remaining tracts in 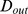 must be assigned to their individual, most similar prototype clusters of 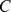, the similarity for each tract 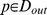 to all prototype representatives in 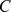 has to be computed by employing the same similarity measures as in stage 2.

The 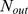 tracts of 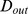 are partitioned into 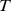 segments of approximately equal size 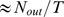. The segments are assigned to 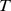 threads and similarity computations are carried out in parallel.

## Supporting Information

Glossary S1 **Glossary that provides short explanations for frequently used terms and abbreviations.**
(PDF)
