# Supplementary material for: Atlas-Guided Cluster Analysis of Large Tractography Datasets
Source: PLoS One. 2013 Dec 30;8(12):e83847. doi: 10.1371/journal.pone.0083847 (PMC3875498; doi:10.1371/journal.pone.0083847)
Supplement: Glossary S1 — Glossary that provides short explanations for frequently used terms and abbreviations. (PDF) [file pone.0083847.s001.pdf]

Glossary

Atlas: A volume-based segmentation of the grey or white matter that divides the brain into separate regions. The atlas resides on 3D Cartesian grid.

Atlas class: A class of the atlas is a specific region in the atlas.

Atlas space: The space in which the atlas resides.

ANTs – Advanced Normalization Tools: A software library used to perform the coregistration of medical images. It has been developed by Avants et al. [1].

Chaining: A phenomenon in which separated clusters with noise elements in between tend to be joined together [2].

CURE – Clustering Using REpresentatives: A cluster analysis algorithm for the clustering of huge databases [3].

$d(p,q)$: The distance function $d(p,q)$ determines the similarity between two tracts p and q on the basis of their distinguishable properties. The function is symmetric, positive semidefinite and reflexive.

Centroid: The centroid for a set of points is the average mean of all points in the set. If the set is ordered and the points are not uniformly distributed along the path described by the set, the segment length between two successive points has to be taken into account during the computation of the centroid.

Cluster: A cluster defines a set of fiber tracts with at least one fiber tract. Tracts in the cluster are similar according to the similarity measure that was used to create the cluster.

CD – Combined Distance: A similarity measure that uses distinct features of the tracts (spatial location, orientation and shape) to determine their similarity.

Distance function: A distance function computes the similarity between two elements on basis of distinguishable features or properties of these elements.

DTI – Diffusion Tensor Imaging: An MRI method that acquires diffusion weighted imaging data in multiple orientations to reconstruct the diffusion tensors.

FDT – Fiber bundle Driven Techniques: Various methods that utilize fiber tracts to perform a quantitative analysis of diffusion weighted MRI data [4–7].

FMRIB58 template: An FA template in MNI152 space that was generated by averaging 58 different, coregistered FA datasets [8].

GM - Grey Matter: The part of the brain and the spinal cord that contains the bodies of nerve cells.

Hierarchical clustering: A set of clustering methods that organize the data into a tree-like hierarchical structure based on the similarity matrix [9]. The resulting cluster tree can be cutted at certain levels to obtain the final clustering.

HAC – Hierarchical Agglomerative Clustering: Hierarchical clustering in which the cluster tree is generated with a bottom up approach.

HD – Hausdorff Distance: $\mathrm{HD}(p,q)$ denotes the Hausdorf distance between two sets of points p and q.

IRQ – Inter-Quartile Range: The difference between the upper and lower quartile (i.e. first and third quartile). In a box plot the lower and upper parts of the (colored) box correspond to the first and third quartile.

k-NNs – k-Nearest Neighbors: For one element p of a dataset, the k-NNs are the k most similar elements to p.

LOF – Local Outlier Factor: The $\mathrm{LOF}(p)$ is a score for element p that specifies the outlierness p with respect to the surrounding elements of p. LOFs were introduced by Breunig et al. in 2000 [10].

Medoid: For a set of elements (e.g. tracts), the medoid is one element of the set that is most similar to all other elements.

Native space: The space of a dataset in which its data was acquired.

Outlier: An outlier is an element of a dataset that has no or low resemblance to other elements of the dataset.

$\phi_A(v_i)$: The probability that voxel v_i belongs to the atlas class A.

Prototype bundles/cluster: The prototype bundles are a set of clusters that are generated during the cluster analysis with CATSER. The prototype bundles are generated from a reduced sample of the whole datasets and are later utilized to assign remaining, unprocessed fiber tracts to their most similar prototype.

Rasterizing: The process of rasterization transfers real valued data to a Cartesian grid.

Representatives: The representatives are a set of well scattered tracts that are distributed throughout a cluster to approximate the shape of the cluster.

$\rho_A(v_i)$: The tract density $\rho_A(v_i)$ corresponds to the number of tracts from bundle A that occupy voxel v_i.

Similarity matrix: A matrix that contains the pair-wise similarities for all elements of a dataset. The pair-wise similarities are computed by utilizing a distance function.

Similarity measure: See distance function.

Spatial agreement: The spatial agreement s rates the correspondence between rasterized tracts and an atlas class according to the number of voxels of the tracts as well as the number of intersecting voxels between tracts and atlas.

Tract: A fiber tract is an ordered set of points in 3D space with at least two points.

Tract-based Spatial Statistics: A method for the quantitative analysis of diffusion weighted MRI data, introduced by Smith et al. in 2006 [11].

TCD – Tract Centroid Distance: The $\mathrm{TCD}(p, q)$ is the Euclidean norm between the centroids of two tracts p and q.

Voxel-Based Morphometry: A method for the quantitative analysis of MRI data, introduced by Ashburner and Friston in 2000 [12].

$w(C_1, C_2)$: The weighting factor $w(C_1, C_2)$ weights the distances between two clusters C_1 and C_2 according the spatial agreement between clusters and atlas classes. This incorporates spatial information of a white matter atlas into the clustering.

WM – White Matter: The part of the brain and the spinal cord that contains the axons of the neurons. Axons (inter-) connect nerve cells and distinct brain regions.

$\zeta(A, C)$: The matching value $\zeta(A, C)$ reflects the spatial resemblance between an atlas class A and a cluster C.

References

1. Avants BB, Tustison NJ, Song G, Cook PA, Klein A, et al. (2011) A reproducible evaluation of ANTs similarity metric performance in brain image registration. Neuroimage 54: 2033–2044.

2. Everitt BS, Landau S, Leese M, Stahl D (2011) Cluster Analysis. Wiley Series in Probability and Statistics.

3. Guha S, Rastogi R, Shim K (2001) Cure: An Efficient Clustering Algorithm for Large Databases. Inf Syst 26: 35-58.

4. Fillard P, Gilmore J, Piven J, Lin W, Gerig G (2003) Quantitative analysis of white matter fiber properties along geodesic paths. Medical Image Computing and Computer-Assisted Intervention-MICCAI 2003 : 16–23.

5. Gong G, Jiang T, Zhu C, Zang Y, Wang F, et al. (2005) Asymmetry analysis of cingulum based on scale-invariant parameterization by diffusion tensor imaging. Hum Brain Mapp 24: 92–98.

6. O'Donnell LJ, Westin CF, Golby AJ (2007) Tract-based morphometry. Med Image Comput Comput Assist Interv 10: 161–168.

7. Ros C, Güllmar D, Stenzel M, Mentzel HJ, Reichenbach JR (2012) Quantitative fiber bundle-based analysis of diffusion-weighted MRI data. Biomed Tech (Berl) 57: 530–533.

8. Jenkinson M, Beckmann CF, Behrens TEJ, Woolrich MW, Smith SM (2012) FSL. Neuroimage 62: 782–790.

9. Xu R, Wunsch DC (2008) Clustering. Wiley-IEEE Press.

10. Breunig MM, Kriegel HP, Ng RT, Sander J (2000) LOF: Identifying Density-Based Local Outliers. In: Chen W, Naughton JF, Bernstein PA, editors, SIGMOD Conference. ACM, pp. 93-104. URL http://doi.acm.org/10.1145/342009.335388.

11. Smith SM, Jenkinson M, Johansen-Berg H, Rueckert D, Nichols TE, et al. (2006) Tract-based spatial statistics: voxelwise analysis of multi-subject diffusion data. Neuroimage 31: 1487–1505.

12. Ashburner J, Friston KJ (2000) Voxel-based morphometry – the methods. Neuroimage 11: 805–821.
